# Physiological and multi-omics insights into ultraviolet B-induced stress adaptation in *Fritillaria cirrhosa* native to the Qinghai-Tibet Plateau

**DOI:** 10.1016/j.jare.2025.10.049

**Published:** 2025-10-26

**Authors:** Zemin Yang, Dan Gao, Ye Wang, Haitao Liu, Yuhan Wu, Haobo Zhang, Haiqing Wang, Xusheng Gao, Jialu Wang, Yonggang Wang, Huigan Xie, Shaobing Fu, Xiwen Li

**Affiliations:** a State Key Laboratory for Quality Ensurance and Sustainable Use of Dao-di Herbs, Institute of Chinese Materia Medica, China Academy of Chinese Medical Sciences, Beijing 100700, China; b State Key Laboratory of Phytochemistry and Natural Medicines, Kunming Institute of Botany, Chinese Academy of Sciences, Kunming 650201, China; c Institute of Traditional Chinese Medicine Health Industry, China Academy of Chinese Medical Sciences, Nanchang 330115, China; d Institute of Medicinal Plant Development, Chinese Academy of Medical Sciences & Peking Union Medical College, Beijing 100193, China; e College of Chinese Medicinal Materials, Jilin Agricultural University, Changchun 130118, China; f School of Life Science and Engineering, Lanzhou University of Technology, Lanzhou 730050, China; g Nin Jiom Medicine Manufactory (Hong Kong) Limited, Hong Kong 999077, China

**Keywords:** *Fritillaria cirrhosa*, Transgenerational stress memory, Adaptive evolution, Transcriptional regulation, Biosynthetic pathway

## Abstract

**Introduction:**

*Fritillaria cirrhosa* (Liliaceae), an endangered medicinal plant of the Qinghai-Tibet Plateau, exhibits strong adaptability to ultraviolet B (UV-B) radiation during the transition from wild to artificial habitats. However, the molecular and physiological mechanisms underlying these adaptive responses remain largely unexplored, which hinders efforts to enhance their conservation and cultivation.

**Objectives:**

The aim of this study was to comprehensively examine the morphological, cellular, and physiological responses of cultivated *F. cirrhosa* to UV-B radiation, with a particular focus on uncovering the key defense mechanisms that confer enhanced plant resilience.

**Methods:**

To simulate its native high-altitude habitat, we augmented the growing area with ultraviolet radiation. Wild type *F. cirrhosa* was used as a control to systematically evaluate the response of cultivated plants to UV-B radiation, focusing on their morphology, cytology, and physiology. Multi-omics analyses were employed to uncover the transcriptional regulatory networks governing the responses of cultivated plants to UV-B stress.

**Results:**

UV-B radiation induced substantial microstructural damage to the leaf mesophyll, chloroplasts, and stomatal complexes in both wild type and cultivated *F. cirrhosa*. However, the cultivated type exhibited significantly enhanced ability to maintain reactive oxygen species (ROS) homeostasis, effectively activating the antioxidant defense system and promoting the biosynthesis of UV-absorbing metabolites—thereby ensuring superior photosynthetic performance under UV-B stress. Mechanistically, upon UV-B detection, UVR8 photoreceptors in cultivated *F. cirrhosa* rapidly interact with the light regulatory protein COP1, forming the UVR8-COP1 complex, which stabilizes the light-signal transcription factor HY5 and modulates the activity of the FcMYB and FcWRKY transcription factors. This regulatory cascade promotes the biosynthesis of flavonoids and lignin, enhancing the transcription of key genes, namely *FcPAL*, *Fc4CL*, *FcC4H*, *FcCHS*, and *FcF3H*. This results in the accumulation of key phenolic compounds (xanthohumol, chrysin, luteolin, galangin, and pinocembrin) that function as “sunscreens” and “ROS scavengers”, thereby effectively reducing UV-B-induced oxidative stress.

**Conclusion:**

Compared to the wild type *F. cirrhosa*, the cultivated type demonstrated heightened UV-B tolerance through the activation of the UVR8-COP1-HY5 signaling pathway and increased biosynthesis of phenolic compounds. These insights provide a strong foundation for breeding strategies and conservation efforts to safeguard this valuable highland species.

## Highlights

• UVR8-COP1 complex regulates target genes for flavonoid and lignin synthesis by stabilising HY5.
• Flavonoid and lignin jointly construct a regulatory network for resistance to UV-B in *F. cirrhosa.*
• Cultivated type *F. cirrhosa* has better UV-B tolerantion than wild type.

## Graphical abstract

**Figure:**
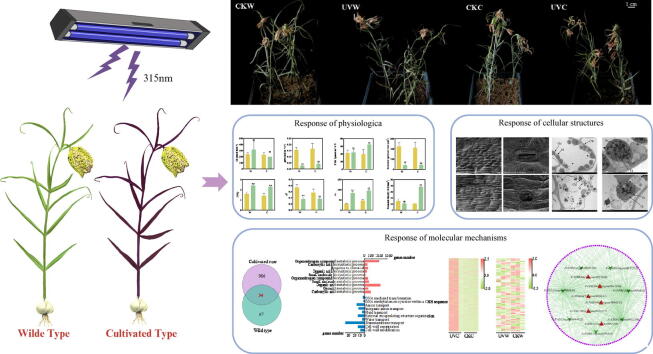
Use the image after the first modification to revise the graphical abstract again.

## Introduction

Human activity, rapid industrialization, and environmental pollution have contributed to the thinning of the ozone layer, resulting in more ultraviolet light reaching the Earth's surface [1]. UV-B radiation, although it constitutes only 0.5 % of the sun’s total radiant energy [2] and only partially reaches the Earth's surface, significantly affects the physiology of plants through light interception in photosynthesis [3]. Owing to smaller optical air masses, UV radiation is more intense at high altitudes, such as in the Tibetan Plateau and the Himalayas [4,5]. Therefore, high-altitude medicinal plants are believed to experience greater UV stress than do low-altitude plants. Enhanced UV-B radiation disrupts plant development, morphology, and physiological functions by increasing the level of reactive oxygen species (ROS), thus upsetting the oxidant-antioxidant balance; and damaging DNA, photosynthetic organs, and cellular structures such as chloroplasts, mitochondria, and stomata [[6], [7], [8]].

Plant species exhibit variable responses to UV-B stress and show a number of adaptive traits to sustain physiological processes such as the wax layer of leaves and trichomes, which limit UV-B penetration [9] and enzymatic and non-enzymatic antioxidants that neutralize free radicals and reduce UV-B damage [10]. UV-B-specific signaling pathways regulate UV protection in plants [11]. The UV resistance locus 8 (UVR8) photoreceptor detects UV-B radiation [12] and interacts with Constitutively Photomorphogenic 1 (COP1) to stabilize Elongated Hypocotyl 5 (HY5), regulating the expression of target genes and promoting the accumulation of UV-protective compounds, such as lignin and phenolics [13,14]. Tolerance and sensitivity to UV-B radiation vary significantly among different plant species and even among phenotypes within the same species [14,15]. For example, Liu et al. [16] found that *A. mongholicus* tolerates UV-B stress by enhancing its antioxidant system, whereas *A. membranaceus* mainly avoids stress by increasing the levels of UV-B-absorbing compounds. Although plant adaptation mechanisms through UV-B radiation regulation have been clarified, their specific regulatory strategies in extremely high-altitude environments remain unclear.

*Fritillaria cirrhosa* (Liliaceae) is a typical high-altitude medicinal plant endemic to the Himalayas and the Hengduan Mountains. It is an important herb used as an antitussive and expectorant and contains unique alkaloids, such as peimine [17,18]. Due to excessive collection, *F. cirrhosa* has been listed as a second-class protected species in China [19]. Wild *F. cirrhosa* is distributed in alpine shrub and meadow ecosystems and primarily displays green phenotypes. However, *F. cirrhosa* exhibits phenotypic plasticity from green to purple when grown in artificial environments and exposed to full sunlight, whereas green phenotypes persist in small quantities. Therefore, we designated the original phenotype retained through domestication as the wild type and the phenotypically plastic phenotype as the cultivated type. Although *F. cirrhosa* is particularly susceptible to UV-B stress owing to the thinner atmosphere of its native habitat and prolonged exposure to sunlight [20,21], cultivated and wild types show marked differences in response to UV-B radiation. Previous studies have revealed that cultivated type exhibit notably elevated alkaloid levels, superior photosynthetic efficiency, and enhanced production of high-quality fruits and seeds relative to their wild type counterparts under unsheltered field conditions. Furthermore, these domesticated types have been found to demonstrate increased resilience in withstanding the extreme meteorological characteristics of high-altitude environments [22]. Given its transition from wild to cultivated environments and its rarity, *F. cirrhosa* serves as a valuable model for studying how high-altitude medicinal plants adapt to UV-B radiation.

This study aimed to screen for genotypes of *F. cirrhosa* with greater resistance to UV-B stress by investigating the differences in photosynthetic capacity, cellular structure, ROS content, antioxidant activity, and UV-absorbing substance content between wild type and cultivated plants under enhanced UV-B radiation, and to uncover the molecular mechanisms of UV-B resistance in *F. cirrhosa* by integrated transcriptomic and metabolomic analyses. Our findings provide an in-depth understanding of molecular drivers, cellular processes, and regulation of secondary metabolites in high-altitude plants as key factors underlying the defense mechanism against UV-B radiation, thus enabling effective screening for medicinal plants with high resistance to UV-B radiation.

## Materials and methods

### Experimental area and soil preparation

The experiment took place in Huzhu Tu Autonomous County, Haidong City, Qinghai Province, China (36°59′E, 101°59′N). The region is situated at an altitude of 3,050 m, with an average daily UV-B radiation of 16.74 kJ/m^2^. The average annual temperature is 0°C, and the average daily temperature reaches 13 °C. Details of the experimental potting soil parameters are provided in the Supplementary Method 1.

### Plant materials

The “lantern-flower” stage represents a pivotal developmental milestone in the reproductive cycle of *F. cirrhosa*, marking the initiation of floral emergence and subsequent fruit formation. *F. cirrhosa* plants were grown under standard field conditions until reaching the lantern-flower stage. At the half-spreading stage, plants with consistent height and stem thickness were transplanted into pots measuring 16 cm in base diameter, 23.5 cm in height, and 22 cm in top diameter. All pots (*n* = 120) contained an equal bulk density of soil and four *F. cirrhosa* plants; half of the pots contained cultivated type *F. cirrhosa* plants, either under UV-B treatment (UVC) (30 pots) or control treatment (CKC) (30 pots). Similarly, the wild type *F. cirrhosa* plants were divided between UV-B treatment (UVW) (30 pots) and (CKW) (30 pots).

### UV-B radiation treatments

**Fig. 1:**
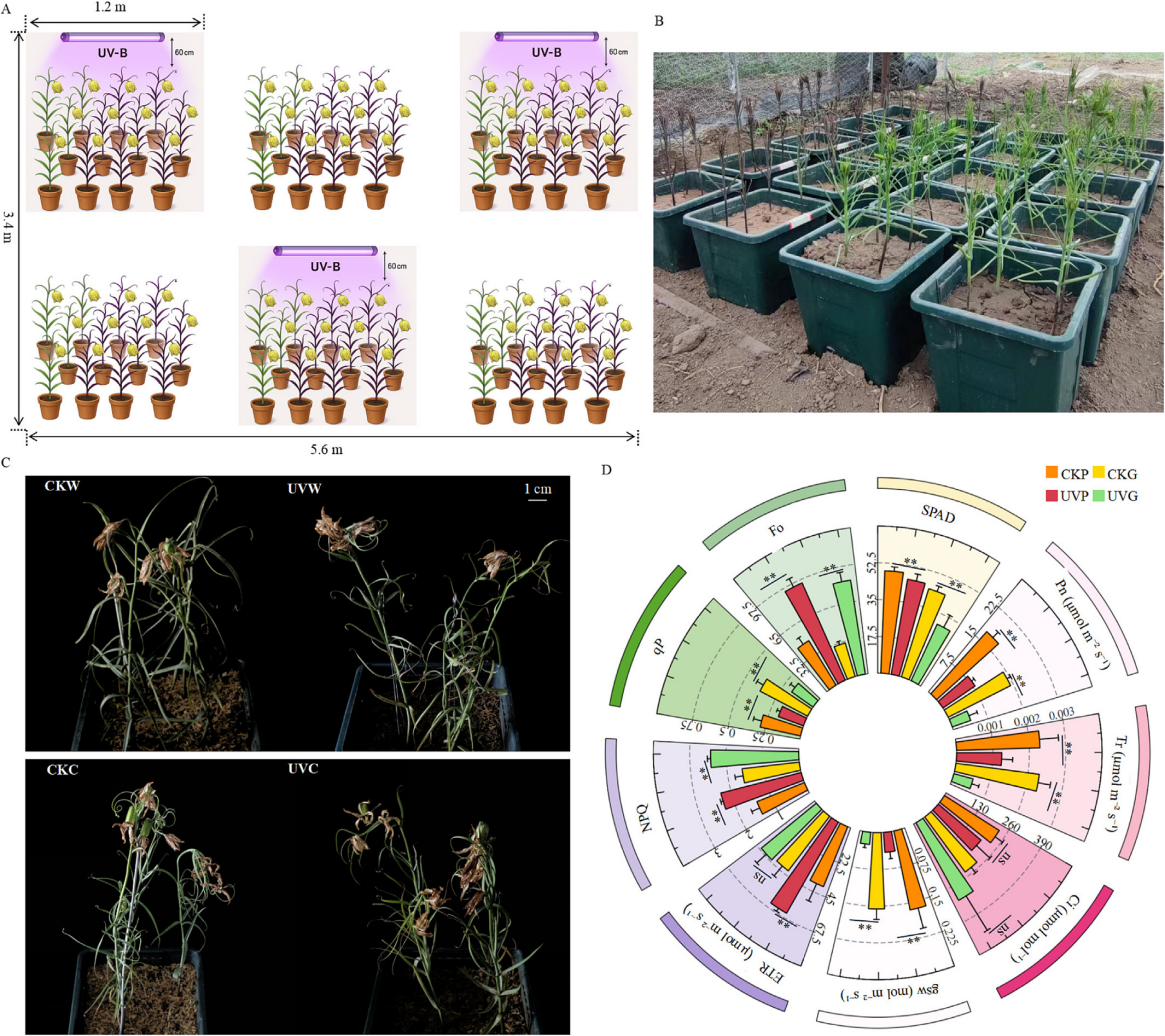
Overview of the experimental design and results. (A) Experimental layout showing 10 pots per type, with red indicating UV-B treatment and green indicating control (CK). (B) Representative close-up image of the plants during UV-B exposure. (C) Phenotypic differences between wild and cultivated *F. cirrhosa* under control and UV-B conditions. (D) Photosynthetic performance of the two types under control and UV-B treatments. Data are presented as Mean ± Se of five biological replicates. Statistical significance: * *P* < 0.05, ** *P* < 0.01, ns, not significant. Treatment abbreviations: CKW, wild type control; UVW, wild type UV-B; CKC, cultivated type control; UVC, cultivated type UV-B.

To simulate the native high-altitude habitat of *F. cirrhosa*, we augmented the natural environment of the Qinghai-Tibet Plateau with ultraviolet radiation using fluorescent lamps (Nanjing Huaqiang Electronics Co., Nanjing, China) with a length of 1,200 mm, diameter of 38 mm, power of 40 W, and wavelength of 313 nm. The distance between the plant canopy and the UV-B lamp was maintained at 60 cm throughout the experiment (Fig. 1A&B). The UV-B irradiation intensity was monitored by a UV irradiometer with λmax = 313 nm (Beijing Shida Photoelectric Technology Co., Ltd., Bejing, China). The irradiation of the UV-B-treated group was 34.56 kJ·m^−2^·d^−1^. The UV-B radiation intensity was set based on an experiment using *Angelica sinensis* [23] and the magnitude of the UV-B increase in the Tibetan Plateau in recent years. The treatment began in July 2023 (base radiation intensity) and lasted 3h per day (11:00 AM to 13:00 PM), with irradiation on sunny days and no irradiation on cloudy or rainy days. Plants treated with UV-B, as well as the CK group, were sampled after 25 days of treatment. In each treatment group, five plants were randomly selected, and three biological replicates of each plant were assessed to determine the photosynthetic fluorescence indices. Fresh leaves were rinsed and used for cytoarchitectural observation and assays of malondialdehyde (MDA), UV-absorbing compounds, ROS, and antioxidants. Remaining tissue was frozen in liquid nitrogen and stored at − 80 °C for further analysis.

### Measurement of photosynthetic parameters

Photosynthesis in *F. cirrhosa* under control and UV-B was assessed using a LI-6800 system (LI-COR, Lincoln, NE, USA). Details on gas exchange and fluorescence are in Supplementary Method 2.

### ROS production, enzymatic antioxidants, MDA content and UV-B absorbing compounds

The H_2_O_2_ content, O_2_^–^ content, peroxidase (POD) activity, and MDA content were assayed according to the protocols for commercially available H_2_O_2_ assay kits, O_2_^–^ assay kits, POD assay kits, and MDA assay kits, respectively (Beijing Solarbio Science & Technology Co., Ltd., Beijing, China). The content of flavonoids and total phenols were measured according to the instructions of the Flavonoid and Total Phenol Kit. (Adsbio, Yancheng, China).

### *F. Cirrhosa* leaf scanning structure, ultrastructure analysis

The cellular and stomatal ultrastructures of *F. cirrhosa* leaves were examined using an HT7800 Biological transmission electron microscope (TEM) and a Regulus 8100 Bio-scanning electron microscope (SEM) (HITACHI Ltd., Japan), respectively. The detailed experimental methods are provided in the Supplementary Data 3. Stomatal characteristics were analyzed using ImageJ software.

### RNA extraction and transcriptome sequencing

Transcriptome sequencing was carried out on the Illumina Novaseq 6000 sequencing platform by Guangzhou Kidio Biotechnology Co. Ltd. (Guangzhou, China). The detailed experimental methods are provided in the Supplementary Method 4. Differentially expressed genes (DEGs) between CK and UV-B groups were identified using edgeR with FDR < 0.05 and |log_2_FC| > 1.

### Metabolomic analysis of *F. Cirrhosa*

Metabolomic analysis was performed by Gene Denovo (Guangzhou, China) using high-performance liquid chromatography-tandem mass spectrometry (LC-MS/MS) (Supplementary Fig. 1). The specific instrumental parameters are shown in the Supplementary Method 5. Different accumulated metabolites (DAMs) were identified using orthogonal partial least squares discriminant analysis (OPLS-DA, VIP ≥ 1) and T-test (*P* < 0.05) between comparison groups.

### Omics integration and visualization

Transcriptomic and metabolomic variables showing statistically significant differences under at least one condition were identified using ANOVA (*P* < 0.01). The two datasets were integrated through the Pearson correlation algorithm in Metware Cloud. To ensure robustness, genes with low correlations to metabolites were excluded by applying correlation (≥0.8) and significance (*P* < 0.05) thresholds for genes and metabolites, respectively [24]. Finally, gene-metabolite correlation networks were constructed using Cytoscape (v3.10.2).

### Weighted gene co − expression network analysis (WGCNA)

After filtering out genes with expression values lower than 19, a total of 11,917 genes were retained for downstream analysis. WGCNA was performed using the WGCNA package (v1.69) in R [25]. A soft-thresholding power (β) of 8 was selected to ensure a scale-free topology with a gene correlation coefficient of 0.75 and an average connectivity of 284.887. Modules were defined with a minimum module size of 300 genes. Module eigengenes were subsequently correlated with key metabolites to construct gene–metabolite association networks. The resulting co-expression networks were visualized using Cytoscape (v3.10.2), and hub genes within modules were identified based on degree centrality.

### Gene expression analysis by quantitative real-time PCR (RT-qPCR)

The cDNA samples were synthesized with 2.0 μg total RNA using the PrimeScript™ RT reagent Kit with gDNA Eraser (Takara, Dalian, China) following the manufacturer’s instruction. The synthesized cDNA was used as the template for RT-qPCR. Using the GAPHD gene as an internal control, samples under both types of control and UV-B radiation were determined using previously described methods, and each sample was analyzed in three independent biological replicates [22]. Primer sequences for the target and internal reference genes are listed in Supplementary Table 1.

### Ethics statement

All the experiments does not involve animal experiments or clinical trials, so there are no ethical issues.

### Statistical analysis

Each experiment was conducted with at least three independent biological replicates (*n* ≥ 3). Results are expressed as mean ± standard deviation (SD). Student's *t*-test assessed significance. For multiple group comparisons, one-way ANOVA followed by Tukey's test was used. Statistical tests were performed using GraphPad Prism (version 9.0.0). Correlated heat maps were generated using TBtools [26]. Circular and bubble charts were generated using the OmicShare online tool (https://www.omicshare.com/tools/) [27].

## Results

### UV-B radiation significantly effects photosynthesis in wild and cultivated type *F. Cirrhosa*

UV-B radiation significantly inhibited the growth and development of both wild and cultivated type plants compared to the CK group, as evidenced by the yellowing and wilting of the leaves (Fig. 1C), but the extent of inhibition is variable at the physiological, cellular, and molecular levels. The soil and plant analyzer development (*SPAD)* index of the wild type was significantly reduced by 32.51 % (*P* < 0.01) under UV-B radiation, whereas that of the cultivated plants did not show a significant difference before and after treatment. UV-B radiation markedly reduced the photosynthetic capacity of both wild and cultivated type plants. Specifically, the gas exchange parameters net photosynthetic rate (*Pn*), transpiration rate *(Tr)* and stomatal conductance (*gsw*) decreased by 72.14 %, 76.33 %, and 84.12 %, respectively, in the wild type, and by 53.29 %, 45.85 %, and 75.33 %, respectively, in the cultivated type (*P* < 0.01). Interestingly, intercellular CO_2_ concentration (*Ci*) did not differ significantly before and after UV-B treatment in the wild and cultivated type plants. The chlorophyll fluorescence parameter photochemical fluorescence quenching coefficient (*qP*) decreased by 51.30 % and 35.27 % (*P* < 0.01) in the wild and cultivated types, respectively, whereas original fluorescence yield (*Fo*) and non-photochemical quenching coefficient (*NPQ*) increased by 171.90 % and 53.26 % (*P* < 0.01) in the wild and by 103.77 % and 62.02 % (*P* < 0.01), respectively. Notably, we found no significant difference in electron transfer rate (*ETR*) before and after treatment of the wild type, but a significant increase of 62.38 % (*P* < 0.01) was observed after treatment of the cultivated type. These findings demonstrate that UV-B radiation reduces chlorophyll content and photosynthetic capacity in both types of *F. cirrhosa*, with the wild type being more adversely affected than the cultivated type (Fig. 1D).

### UV-B radiation-induced damage to the stomatal structure of leaves in wild and cultivated type *F. Cirrhosa*

**Fig. 2:**
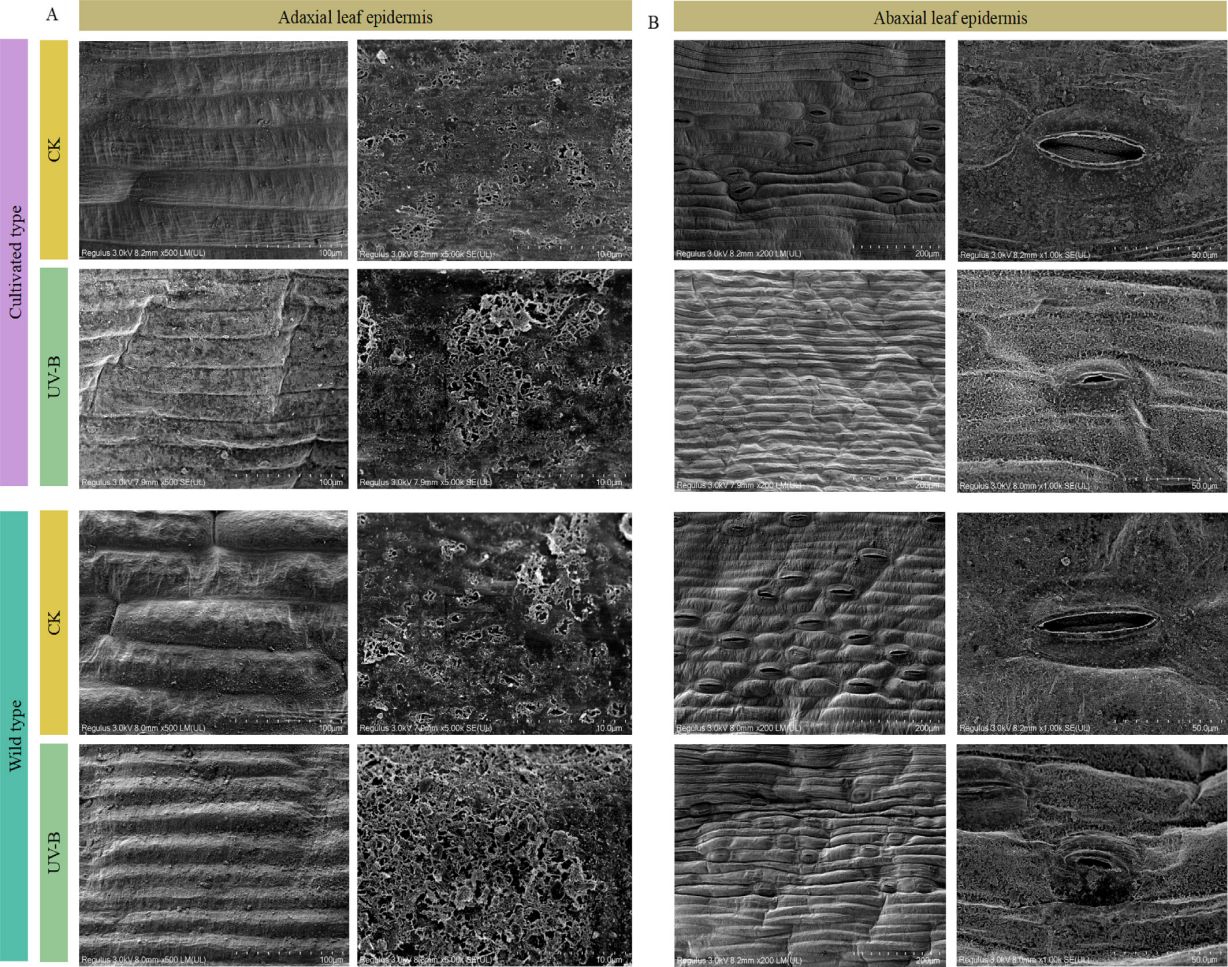
Effect of UV-B radiation on the scanning structure of two types *F. cirrhosa* leaves. (A) Adaxial leaf epidermis without stomata, showing a marked increase and breakage of crystalline particles under UV-B stress, with breakage more pronounced in the wild type. (B) Abaxial leaf epidermis with stomata, where UV-B caused closure, shrinkage; stomatal density decreased in the wild type but increased in the cultivated type.

To investigate the micromorphology of the leaf epidermis in the wild type and cultivated plants under UV-B radiation, we performed SEM. In the adaxial epidermal structure of leaves from the wild and cultivated types, no stomatal distribution was observed in either the UV-B stress or CK groups. However, a small number of crystalline granular attachments were noted in the CK group of both the wild and cultivated types. Under UV-B stress, these attachments increased significantly and exhibited noticeable breakage,and the degree of breakage was significantly greater in the wild type than in the cultivated type (Fig. 2A). We hypothesized that these attachments consist of wax and other metabolic substances.

**Fig. 3:**
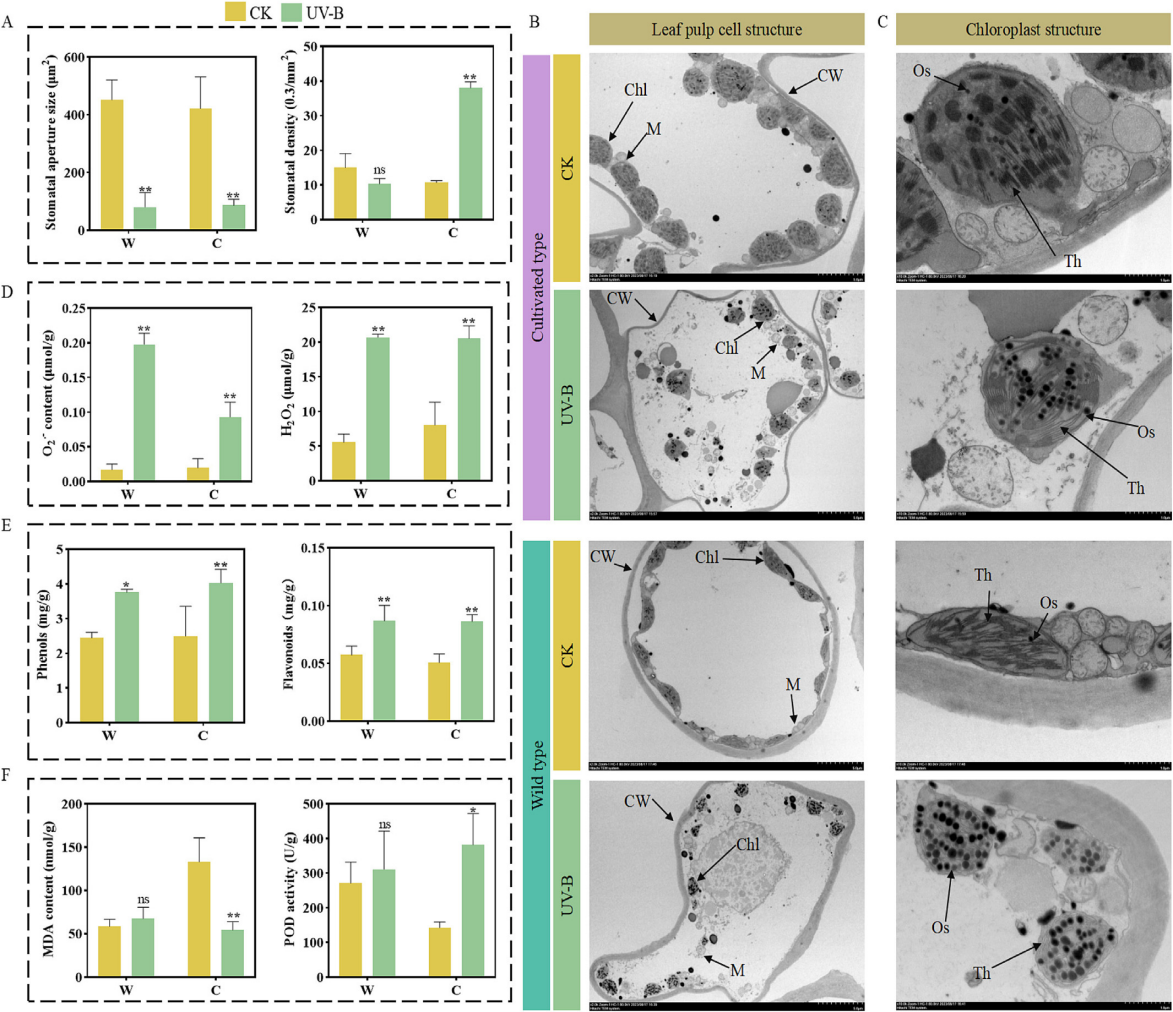
Effect of UV-B radiation on physiological indices and ultrastructure of leaves of two types *F. cirrhosa*. (A) stomatal aperture size and stomatal density. (B) Leaf pulp cell structure. (C) Chloroplast structure, Chl-chloroplasts, M−mitochondria, Th-thylakoid, CW-cell wall, Os-osmiophilic granules. (D) O_2_^–^ and H_2_O_2_ content. (E) Phenols and flavonoids content. (F) MDA content and POD activity. Physiological indices are expressed as Mean ± Se of three biological replicates and the stomatal index are 20 biological replicates, * *P <* 0.05, ** *P <* 0.01.

The abaxial epidermis of the leaves, which contain stomata, revealed distinct differences between the CK and UV-B-treated groups. In the CK group, the stomata of the wild and cultivated types were narrowly elliptical, open, and irregularly distributed, with the stomata slightly protruding relative to the epidermal cells. Conversely, the stomata of the UV-B-treated leaves showed evident damage and shrinkage, with most stomata appearing closed and invaginated. A few stomata remained open, but to a much lesser degree, and there was a noticeable increase in the crystalline particles surrounding the stomata (Fig. 2B). Further measurements revealed that the stomatal aperture size was significantly reduced by 82.09 % and 79.52 % in the wild and cultivated types, respectively. Interestingly, although stomatal density decreased by 45.16 % in the wild type plants, it significantly increased by 71.93 % in the cultivated plants under UV-B stress (Fig. 3A).

### UV-B radiation caused damage to the leaf pulp and chloroplasts structure of leaves of wild type and cultivated *F. Cirrhosa*

To investigate the extent of damage, we used TEM to observe the leaf tissues of the wild and cultivated type plants under UV-B radiation at different magnifications (Fig. 3B and C). The mitochondria, chloroplasts, and other organelles in the leaves of the CK group of the both types of *F. cirrhosa* were clearly structured; the plasma membrane of the protoplasts was smooth and intact; the chloroplasts were compactly arrayed and ellipsoidal or shuttle-shaped; and the osmiophilic granules were of uniform electron density. The protoplasts of the leaf pulp cells of both types of *F. cirrhosa* showed varying degrees of deformation and compression, plasma membrane damage or breakage, organelle disintegration, and slight crumpling of the cell wall after UV-B stress. As well, the chloroplasts were reduced in number and were found to have gradually swelled and broken up, becoming spherical in shape and containing a large concentration of osmiophilic particles, as well as having a high electron density. This indicates that the leaf pulp cells and chloroplast structures of the wild and cultivated types were significantly damaged under UV-B stress; however, compared with the cultivated type, the chloroplast grana and stroma of the wild type had obscure boundaries with loose structures, and the stroma-thylakoid was significantly fractured.

### UV-B radiation induces differential antioxidant responses in wild type and cultivated *F. Cirrhosa*

A comprehensive multiparameter analysis was conducted to elucidate the physiological and biochemical mechanisms of *F. cirrhosa*’s antioxidant response to UV-B radiation. This involved the quantification of ROS accumulation, antioxidant enzyme activity, and the biosynthesis of UV-absorbing in both the wild and cultivated type forms of the plant. Exposure to UV-B radiation significantly increased ROS levels in both wild and cultivated type *F. cirrhosa* relative to ROS levels in in the CK groups. Specifically, the H_2_O_2_ content in wild type and cultivated plants increased by 269.76 % and 157.68 %, respectively, whereas the superoxide anion content increased by 1065.34 % and 371.08 %, respectively (*P* < 0.01) (Fig. 3D). Additionally, the levels of UV-absorbing compounds, such as flavonoids and phenolic compounds, were significantly elevated in the wild type and cultivated type plants under UV-B radiation compared to the corresponding results in the CK group (Fig. 3E). The flavonoid content increased by 51.72 % and 70.52 % (*P* < 0.01), whereas the phenolic content increased by 54.08 % (*P* < 0.05) and 61.59 % (*P* < 0.01) in the wild and cultivated types, respectively. These results indicate that the cultivated type exhibited a significantly greater increase in these compounds than the wild type. In addition, the activity of the antioxidant enzyme POD significantly increased by 167.99 % in the cultivated type compared to that in the CK under UV-B radiation conditions (*P* < 0.05), whereas no significant difference was observed in the wild type. To assess the impact of UV-B radiation on membrane lipid peroxidation in wild type and cultivated plants, we measured the MDA content. Interestingly, a significant decrease in MDA content of 59.16 % (*P* < 0.01) was observed in the cultivated type under UV-B radiation, whereas the wild type showed no significant change before and after treatment (Fig. 3F).

### Degs were significantly more abundant in cultivated than in wild type plants under UV-B radiation

To investigate the impact of UV-B radiation on biosynthesis-related gene expression in wild and cultivated type plants, we conducted transcriptome sequencing on CK- and UV-B-treated samples. The sequencing generated 536.8 million clean reads (79.48 Gb) with high quality (Q20 > 95 %, Q30 > 89 %, GC ∼ 50 %), and de novo assembly produced 99,268 unigenes with an average length of 859 bp (Supplementary Tables 2–3). Principal component analysis (PCA) revealed clear separation between CK and UV-B groups in both types, confirming strong transcriptional responses to UV-B (Supplementary Fig. 2A). Interestingly, the cultivated type exhibited a markedly larger response, with 5,563 DEGs (4,714 up-regulated and 850 down-regulated), whereas the wild type showed only 481 DEGs (247 up-regulated and 234 down-regulated) (Supplementary Tables 4–5). Volcano plots and clustering heat maps further highlighted these genotype-specific responses (Supplementary Fig. 2B&C).

### UV-B radiation resulted in greater functional enrichment in the cultivated type than in the wild type

**Fig. 4:**
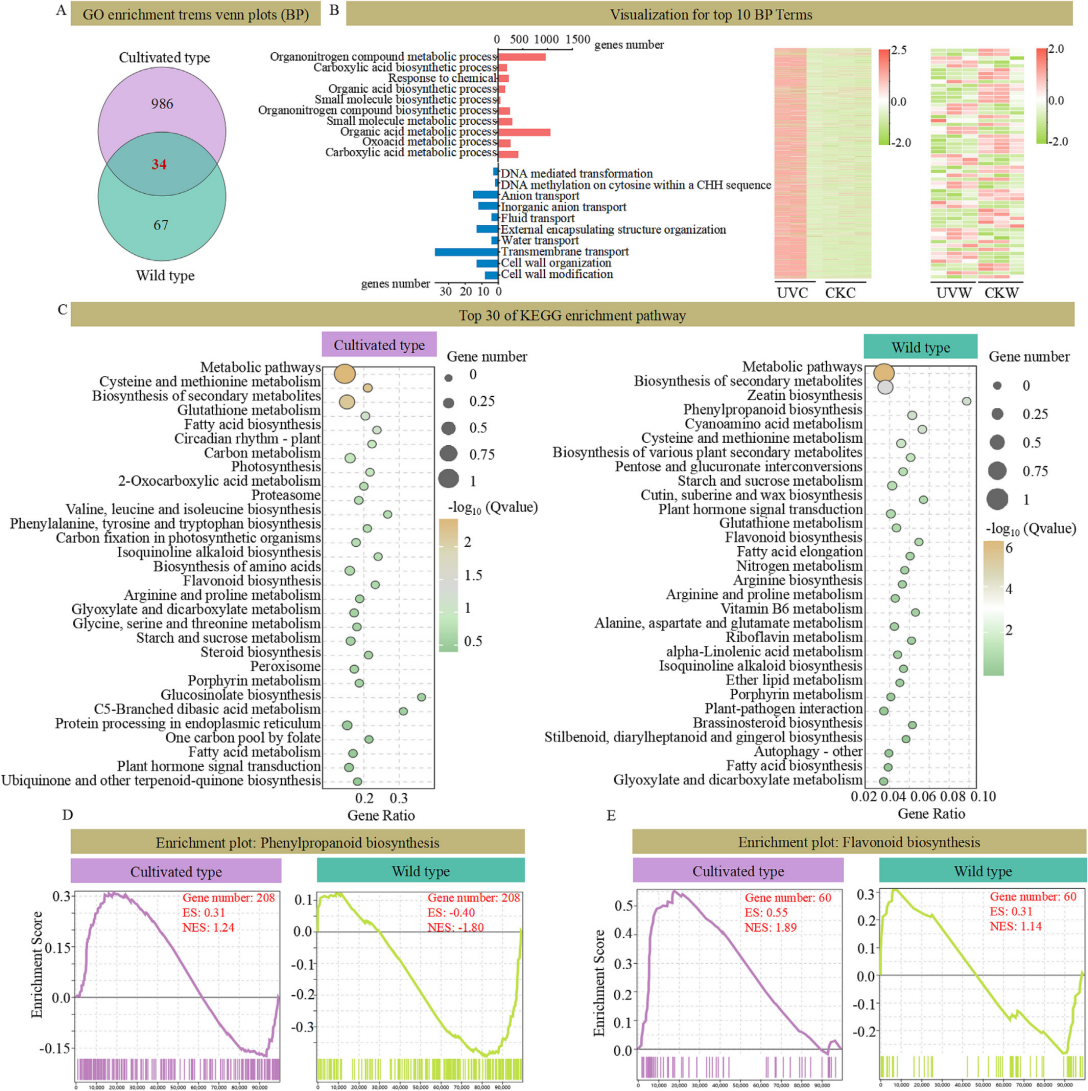
Enrichment analysis of DEGs in wild and cultivated *F. cirrhosa*. (A) Venn diagram showing shared and unique BP terms between the two types. (B) Top 10 enriched BP terms for each type, highlighting distinct functional categories, with the cultivated type showing broader metabolic enrichment and the wild type enriched in cell wall organization. Heat maps visualize the expression of genes associated with these top BP terms. (C) The top 30 KEGG enrichment bubble plot of DEGs in both types *F. cirrhosa.* The horizontal axis represents the rich factor, while the vertical axis represents the pathway names. The color of circles represents the statistical significance of enriched KEGG pathway. The size of the circles represents the number of genes in a KEGG pathway. (D, E) GSEA of the phenylpropanoid and flavonoid biosynthesis pathways, highlighting up-regulation in the cultivated type and weaker or down-regulation in the wild type.

To interpret functional differences between wild and cultivated types, we performed Gene Ontology (GO) enrichment analysis with a significance threshold of *P* < 0.05, focusing on Biological Process (BP) terms. Our results showed that UV-B radiation resulted in greater functional enrichment in the cultivated type than in the wild type. Specifically, a total of 101 BP terms were significantly enriched in the wild type, whereas 1,020 significantly enriched BP terms were identified in the cultivated type (Supplementary Table 6A&B). Venn diagram analysis further revealed 34 shared BP terms, 986 unique to the cultivated type, and 67 unique to the wild type (Fig. 4A). Visualization of the top 10 terms showed that the cultivated type was mainly enriched in organonitrogen compound metabolic process, carboxylic acid biosynthetic process, organic acid metabolic process, small molecule biosynthetic process, and related categories. In contrast, the wild type was mainly enriched in DNA mediated transformation, DNA methylation on cytosine within a CHH sequence, anion transport, water transport, cell wall organization, and cell wall modification (Fig. 4B). Notably, for the 34 shared terms, the number of DEGs annotated in the cultivated type was substantially greater than in the wild type (Supplementary Fig. 2D).

The biological functions of DEGs under UV-B stress were further examined by KEGG pathway analysis. In the cultivated type, 1,735 DEGs were enriched in 132 pathways, with significant overrepresentation in 24 pathways, while the wild type showed only 124 DEGs mapped to 72 pathways, with 15 reaching significance (*P* < 0.05, Supplementary Tables 7A&B). Although both types showed enrichment in antioxidant and UV-B protective pathways such as starch and sucrose metabolism, glutathione metabolism, and flavonoid biosynthesis, the cultivated type exhibited broader and stronger enrichment trends, particularly in pathways related to primary metabolism and growth regulation, including fatty acid biosynthesis, carbon fixation in photosynthetic organisms, carbon metabolism, photosynthesis, and biosynthesis of amino acids. In contrast, the wild type was primarily enriched in pathways associated with hormone signaling and structural adaptation, such as zeatin biosynthesis, phenylpropanoid biosynthesis, plant hormone signal transduction, cutin, suberine and wax biosynthesis, and fatty acid elongation (Fig. 4C). Interestingly, under UV-B stress, we identified 26 DEGs (only three downregulated) associated with photosynthesis and 11 DEGs (four downregulated) involved in the cutin, suberine, and wax biosynthesis pathway in the cultivated type, whereas in the wild type, only one DEG (downregulated) and three DEGs (two downregulated) were detected in these respective pathways (Supplementary Fig. 3A&B). Combined with KEGG annotation, flavonoid biosynthesis was identified as a commonly enriched pathway in both wild and cultivated types. Therefore, we performed GSEA to examine the transcriptional regulation of upstream genes and the pathway itself. The results showed that revealed that genes in the initial steps of phenylpropanoid biosynthesis were up-regulated in the cultivated type but down-regulated in the wild type, with stronger activation of downstream flavonoid and lignin synthesis and higher NES in the cultivated type (Fig. 4D&E). These findings suggest that the cultivated type more effectively mobilizes phenylpropanoid-derived defenses under UV-B stress.

### Transcriptional regulation by UVR8 and transcription factors(TFs) in wild type and cultivated *F. Cirrhosa*

**Fig. 5:**
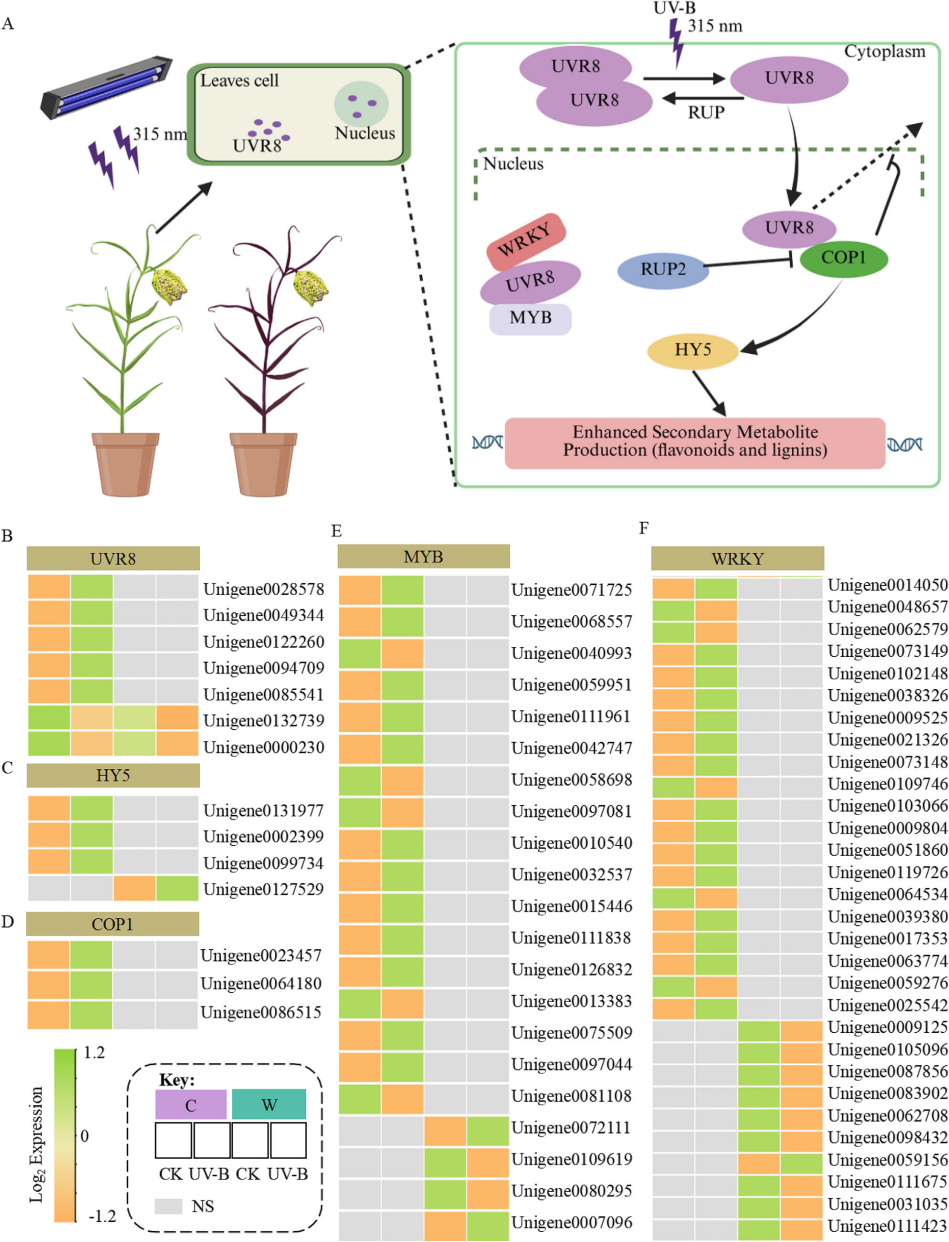
Transcriptional regulation of UV-B responses in wild and cultivated *F. cirrhosa*. (A) Schematic of the UVR8-mediated UV-B signaling pathway, showing interactions with downstream TFs and target genes [[27], [28], [29], [30]]. Heatmap of expression of genes and transcription factors involved in UVR 8 activation. (B) UVR8, UV resistance locus 8. (C) HY5, Elongated Hypocotyl 5; (D) COP1, Constitutively Photomorphogenic 1. (E) MYB family. (F) WRKY family.

UVR8 is the only known photoreceptor in plants capable of sensing UV-B radiation and transmitting UV-B signals [28]. It plays a central role in UV-B photomorphogenesis and stress tolerance by interacting with downstream TFs and target genes [[29], [30], [31], [32], [33]] (Fig. 5A). To explore how this pathway contributes to UV-B responses in *F. cirrhosa*, we screened UVR8-associated genes and TFs (Fig. 5B–D, Supplemental Table 8A). Our analysis revealed a clear divergence between cultivated and wild types. In the cultivated type, seven DEGs encoding UVR8 were detected (five upregulated, two downregulated), whereas only two downregulated DEGs were identified in the wild type. Similarly, three HY5 genes were upregulated in the cultivated type, compared with only one in the wild type. Notably, three upregulated COP1 genes were found exclusively in the cultivated type. Downstream TFs also displayed distinct expression patterns: under UV-B stress, the cultivated type showed strong transcriptional activation, with 12 upregulated and five downregulated FcMYB genes, and 15 upregulated and five downregulated FcWRKY genes. In contrast, the wild type exhibited limited activation, with only two FcMYB genes upregulated (two downregulated) and one FcWRKY gene upregulated (nine downregulated) (Fig. 5E&F, Supplemental Table 8B). These results suggest that the cultivated type maintains a more robust UVR8–HY5–COP1 signaling module and activates a broader set of stress-related TFs, which may underpin its enhanced tolerance to UV-B stress.

### Differences in DAMs and KEGG functional enrichment between cultivated and wild types under UV-B radiation

**Fig. 6:**
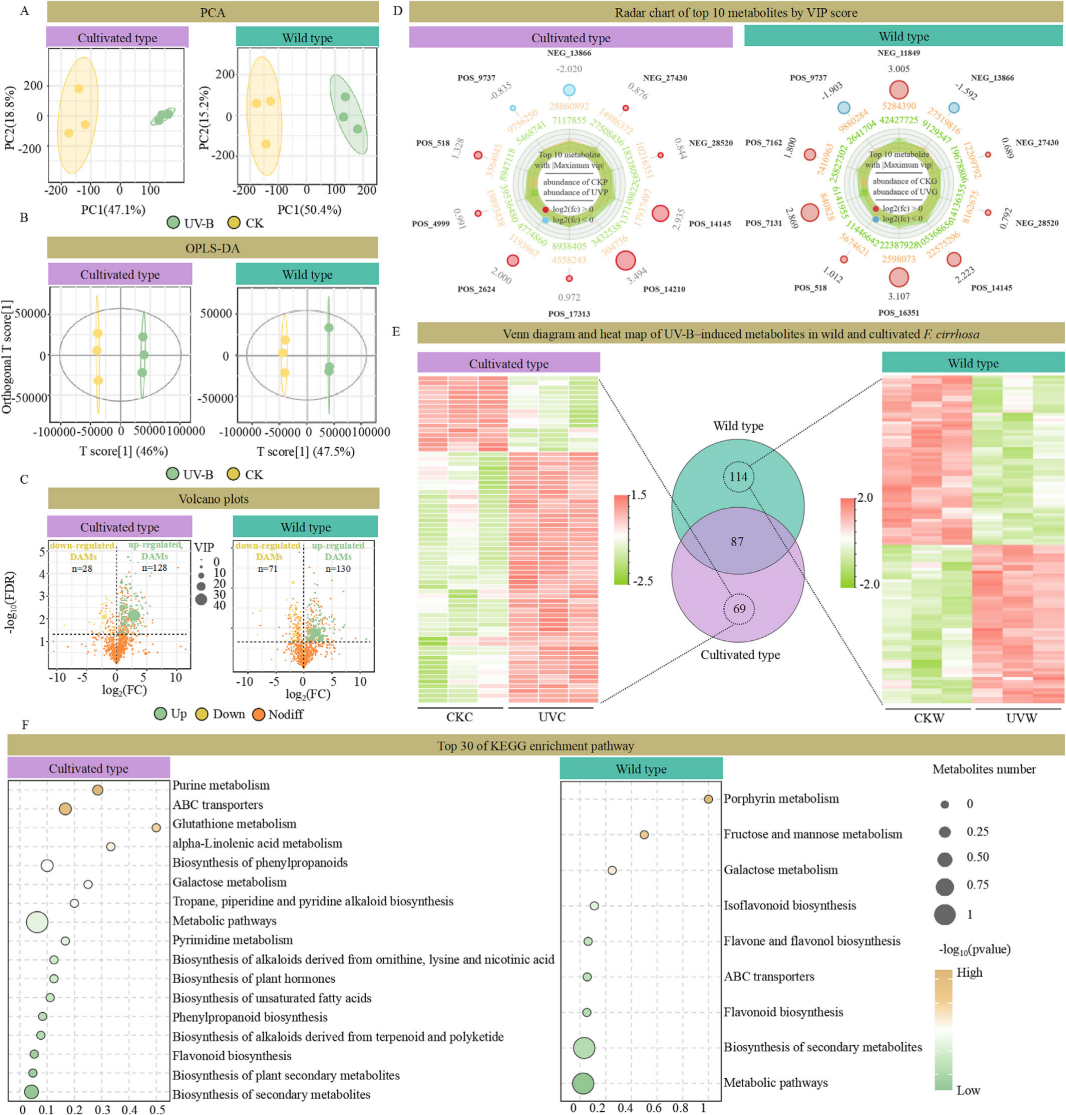
Metabolomic profiling of wild and cultivated *F. cirrhosa* under UV-B stress. (A) Plot of PCA and (B) OPLS-DA scores for all detected metabolites, showing differences between samples based on DAMs. (C) Volcano plots of differential accumulated metabolites (DAMs) in each type. (D) Radar chart of the top 10 metabolites with highest VIP scores, highlighting shared and type-specific metabolites. Venn diagram (E) showing shared and type-specific metabolites, and heat map illustrating UV-B–induced accumulation patterns in wild and cultivated *F. cirrhosa*. (F) KEGG enrichment analysis bubble plots of two type-specific metabolites. The horizontal axis represents the rich factor, while the vertical axis represents the pathway names. The color of circles represents the statistical significance of enriched KEGG pathway. The size of the circles represents the number of genes in a KEGG pathway.

To identify the abundance of differential metabolites in the wild and cultivated types under UV-B radiation, we performed metabolomic analysis using LC-MS/MS. The score plots (Fig. 6A&B) showed a clear separation between the two groups, highlighting significant differences in metabolites between the wild and cultivated types. A total of 156 DAMs were found in the cultivated type (Supplemental Table 9) and 201 in the wild type (Supplemental Table 10). Volcano plots showed that 128 metabolites were up-regulated and 28 were down-regulated in the cultivated type; in the wild type, 130 metabolites were up-regulated and 71 were down-regulated (Fig. 6C). To compare metabolic responses between the wild and cultivated types, we first visualized the top 10 metabolites with maximum VIP values using a radar chart. Six metabolites were shared by both types, while erucamide, dimethachlor, and β-solanine were specific to the cultivated type; delphinidin 3-glucoside, solanidine, enniatin B1, and a cyclopenta [a] phenanthrene derivative were unique to the wild type (Fig. 6D). Venn diagram analysis further showed 87 metabolites shared by both types, 69 unique to the cultivated type, and 114 unique to the wild type. Heatmap visualization revealed that UV-B treatment induced significant accumulation of many type-specific metabolites in both groups (Fig. 6E). KEGG enrichment analysis of two type-specific metabolites revealed distinct patterns. The cultivated type was enriched mainly in nucleotide and energy metabolism (purine and pyrimidine metabolism), detoxification pathways (glutathione metabolism), lipid and hormone metabolism (α-linolenic acid metabolism, biosynthesis of unsaturated fatty acids, biosynthesis of plant hormones), and secondary metabolite biosynthesis (phenylpropanoids, flavonoids, tropane, piperidine, pyridine alkaloids). By contrast, the wild type was enriched in porphyrin metabolism, sugar metabolism (fructose, mannose, galactose), and flavonoid-related pathways (isoflavonoid, flavone, and flavonol biosynthesis) (Fig. 6F).

### Compounds involved in the flavonoid and lignin metabolic pathway undergo dynamic changes in both the cultivated and wild types

**Fig. 7:**
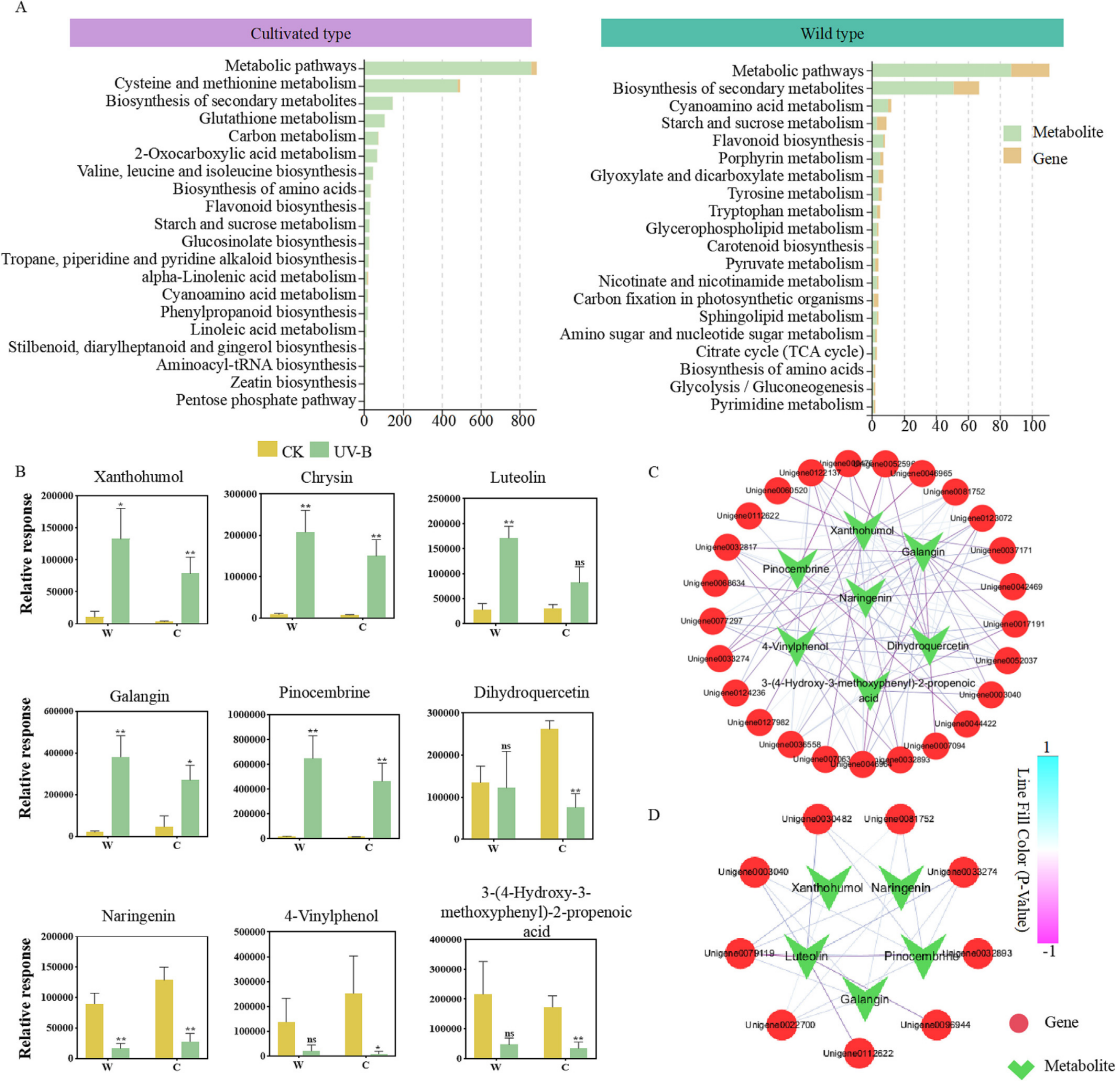
Combined metabolomic and transcriptomic analyses. (A).The top 20 KEGG pathways enriched with metabolomic and transcriptomic. (B) Univariate analysis of the relative abundance of important metabolites involved in lignin and flavonoid biosynthetic pathways, data are expressed as Mean ± SE of three biological replicates. ns: not significant, * *P <* 0.05, ** *P <* 0.01. (C) Correlation network constructed from genes associated with metabolites. Metabolites are indicated by large green rhombus. Genes are indicated by large red triangle.

To characterize the dynamic changes of compounds involved in the flavonoid and lignin metabolic pathways and compare the UV-B stress-induced responses between cultivated and wild types, we analyzed transcriptomic and metabolomic data. The results indicated that compounds involved in the flavonoid and lignin metabolic pathways exhibited dynamic changes in both the cultivated and wild types. UV-B stress promoted the accumulation of flavonoid and lignin compounds in both typess (Fig. 7A & Supplementary Table 11). Specifically, univariate analyses revealed that, compared with the control group, the contents of xanthohumol, chrysin, galangin, and pinocembrine increased sequentially by 1,147.12 %, 2,261.05 %, 1,725.63 %, and 4,143.51 % in the wild types, and by 2,212.38 %, 1,966.50 %, 492.54 %, and 3,520.15 % in the cultivated types, respectively. In contrast, the content of naringenin decreased by 81.59 % in the wild type and 78.30 % in the cultivated type. Interestingly, 3-(4-hydroxy-3-methoxyphenyl)-2-propenoic acid, dihydroquercetin, and 4-vinylphenol exhibited no significant changes in content in the wild type, but decreased by 80.20 %, 71.03 %, and 96.49 % in the cultivated type. Furthermore, the content of lutein increased by 512.92 % in the wild type, whereas no significant change in lutein content was observed in the cultivated type (Fig. 7B). Together, these results highlight pronounced changes in flavonoid and lignin metabolism under UV-B stress, with distinct responses between wild and cultivated plants.

### Flavonoid and lignin correlation with the ROS system

To reveal the link between metabolites in the biosynthetic pathways of flavonoids and lignins and the ROS system under UV-B radiation in wild type and cultivated types, we performed correlation analyses between POD, MDA, O_2_^–^, H_2_O_2_, and DAMs (Supplementary Fig. 4). The results indicated that in the cultivated type, the total flavonoid content was significantly positively correlated with the contents of POD, O_2_^–^ and H_2_O_2_, and significantly negatively correlated with the content of MDA (*P* < 0.05), whereas in the wild type, the total flavonoid content was significantly positively correlated with the contents of O_2_^–^ and H_2_O_2_ (*P* < 0.05). Further analysis of the screened DAMs revealed that in the wild and cultivated types, O_2_^–^ and H_2_O_2_ contents were significantly positively correlated (*P* < 0.05) with xanthohumol, chrysin, galangin, and pinocembrine, and with naringenin; 3-(4-hydroxy-3-methoxyphenyl)-2-propenoic acid showed a significant negative correlation (*P* < 0.05). Interestingly, luteolin was significantly positively correlated (*P* < 0.05) with O_2_^–^ and H_2_O_2_ contents only in the wild strain. The screened DAMs did not present a certain correlation with the content of either POD or MDA in the wild type; the content of MDA showed a significant negative correlation with xanthohumol, galangin, luteolin, chrysin, and pinocembrine, and dihydroquercetin and naringenin were significantly positively correlated with the cultivated type (*P* < 0.05). POD content was also significantly negatively correlated with dihydroquercetin and naringenin in the cultivated type; and chrysin and pinocembrine were significantly positively correlated (*P* < 0.05).

### Comprehensive response network of wild and cultivated type *F. Cirrhosa* under UV-B stress was constructed through transcriptome and metabolome

Because of the significant changes in flavonoid and lignin biosynthesis in wild type and cultivated type under UV-B treatment (Fig. 7A), transcriptomics and metabolomics data were integrated using the Pearson algorithm of Metware Cloud, and correlation networks of gene and metabolite were constructed using Cytoscape (v3.10.2) (Fig. 7C). A total of 25 DEGs and eight metabolites were identified in the cultivated type, and nine DEGs and six metabolites were identified in the wild type based on the thresholds of *r* > 0.8 and *P* < 0.05 (Supplementary Table 12).

### Flavonoid and lignin metabolic pathways participating in the protective effects of UV-B stress

**Fig. 8:**
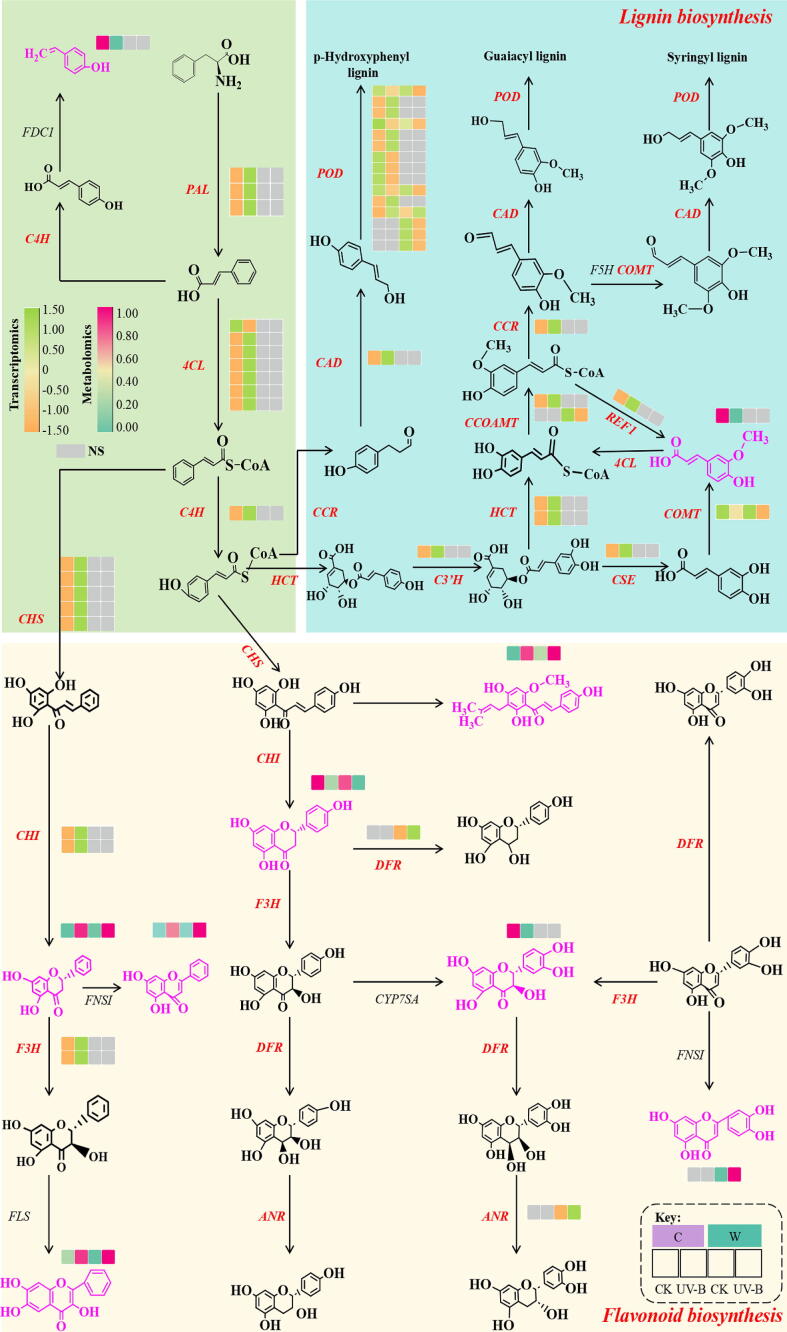
The key pathway analysis, green part is the phenylpropanoid pathway, the blue part is the lignin biosynthesis pathway and the yellow part is the flavonoid biosynthesis pathway. The green boxes on either side of the line indicate up-regulated DEGs and yellow boxes indicate down-regulated DEGs, and the degrees of the colors indicated the expression levels (TPM value) of these genes. The position of the nodes are metabolites. Red colours represent metabolites that were detected, and red boxes on either side of the line indicate up-regulated DAMs and blue boxes indicate down-regulated DAMs.

We therefore reconstructed the flavonoid and lignin metabolic pathways based on metabolites and genes with significant changes (*P* < 0.05) between CK and UV-B treatmentsas (Fig. 8; Supplementary Tables 13&14). Under UV-B stress, in cultivated type, a total of 14 DEGs encoding critical precursor enzymes in flavonoid and lignin biosynthesis, including three phenylalanine ammonia-lyase (*FcPAL*), seven 4-coumarate--CoA ligase (*Fc4CL*), one *trans*-cinnamate 4-monooxygenase (*FcC4H*), two shikimate O-hydroxycinnamoyltransferase (*FcHCT*) and one 5-O-(4-coumaroyl)-D-quinate 3′-monooxygenase (*FcC3′H*). Only one DEG encoding *Fc4CL* was significantly down-regulated, the other 13 were significantly up-regulated. However, no DEGs were found to encode any of the above enzymes in the wild type strain. Interestingly, 4-vinylphenol content also decreased in the cultivated type. For flavonoid biosynthesis, nine DEGs identified in the cultivated type were significantly upregulated, with five DEGs encoding chalcone synthase (*FcCHS*), two DEGs encoding chalcone isomerase (*FcCHI*), and two DEGs encoding naringenin 3-dioxygenase (*FcF3H*); the two DEGs identified in the wild type encoding bifunctional dihydroflavonol 4-reductase (*FcDFR*) and anthocyanidin reductase (*FcANR*). These findings were also accompanied by increased xanthohumol, pinocembrine, chrysin, and galangin content and decreased naringenin content in wild type and cultivated plants. Interestingly, dihydroquercetin was screened only in the cultivated type and its content was also reduced, whereas luteolin was screened only in the wild type and its content was increased. Regarding lignin biosynthesis, 18 (11 up-regulated and seven down-regulated) and nine DEGs (one up-regulated and eight down-regulated) were identified in the cultivated type and wild type, respectively, which involved peroxidase (*FcPOD*), cinnamyl-alcohol dehydrogenase (*FcCAD*), cinnamyl-alcohol dehydrogenase (*FcCCR*), caffeoyl-CoA O-methyltransferase (*FcCCOAMT*), caffeoylshikimate esterase (*FcCSE*), caffeic acid 3-O-methyltransferase (*FcCOMT*) and convenyl-aldehyde dehydrogenase (*FcREF1*). These findings were also accompanied by a decrease in 3-(4-hydroxy-3-methoxyphenyl)-2-propenoic acid content in the cultivated type. A comprehensive analysis of DEGs and DAMs in the flavonoid and lignin biosynthesis pathways of the wild and cultivated types revealed that UV-B stress positively affected both pathways. However, the screened DEGs and DAMs were more abundant in the cultivated type, and their expression and content were higher than those in the wild type, indicating that flavonoids and lignin compounds were more likely to accumulate in the cultivated type under UV-B stress. Three genes involved in flavonoid biosynthesis, *FcCHS* (Unigene0007094), *FcF3H* (Unigene0103205), *FcCHI* (Unigene0037171), and FcMYB (Unigene0059951) of the MYB TFs family, were validated by RT-qPCR (Supplementary Fig. 5). These results aligned with the RNA-seq data, supporting the reliability of the transcriptome analysis.

### Identification of key transcription factors involved in the biosynthesis of flavonoids and their upstream phenylpropanoid metabolites

**Fig. 9:**
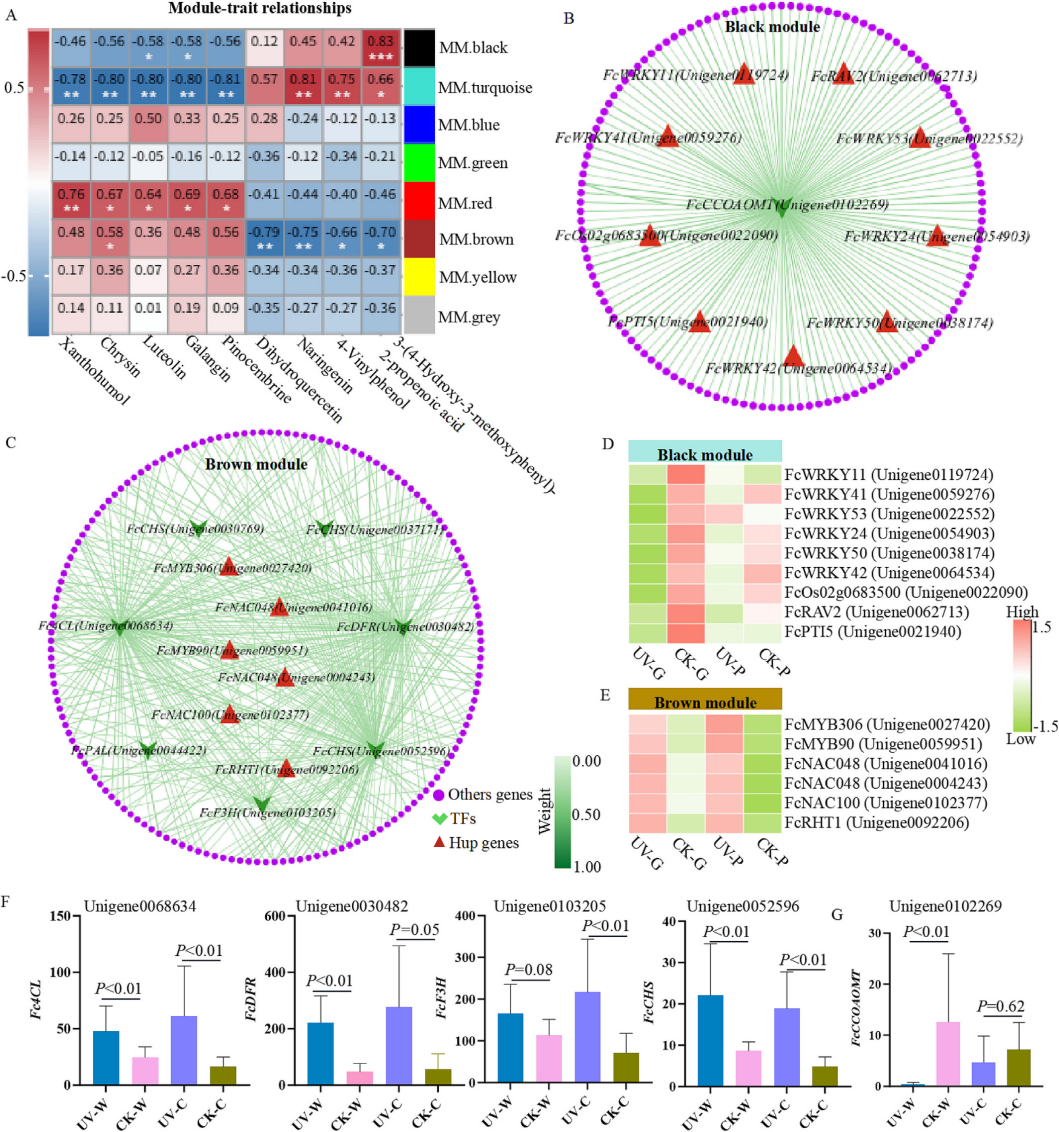
Identification of key TFs associated with lignin and flavonoid biosynthesis in *F. cirrhosa*. (A) Module-trait relationships, negative numbers (blue) indicate that the characterised genes are negatively correlated with the module, and positive numbers (red) indicate positive correlation. (B-C) Associated networks of differentially expressed structural genes and TFs involved in lignin and flavonoid biosynthesis. (D-G) Expression patterns of genes and TFs in the black and brown modules, showing downregulation in the black modules and upregulation in the brown module.

To investigate the regulatory mechanisms of *F. cirrhosa* during growth and development in response to UV-B radiation, we employed the WGCNA package to identify key modules associated with the accumulation of flavonoids and their upstream phenylpropanoid metabolites. Co-expression networks were subsequently constructed based on the key modules identified by WGCNA (Fig. 9A, Supplementary Fig. 6A&B, Supplementary Table 15). Within the black module, we detected a critical gene, *FcCCOAOMT* (Unigene0102269), together with several important transcription factors, including FcWRKY11 (Unigene0119724), FcWRKY41 (Unigene059276), FcWRKY42 (Unigene0064534), FcWRKY53 (Unigene0022552), FcWRKY24 (Unigene0054903), FcWRKY50 (Unigene0038174), FcPTI5 (Unigene0021940), FcRAV (Unigene0062713), and FcOs02g0683500 (Unigene0022090) (Fig. 9B). The brown module contained another set of transcription factors, such as FcMYB306 (Unigene0027420), FcMYB90 (Unigene0059951), FcNAC048 (Unigene0041016; Unigene0004243), FcNAC100 (Unigene0102377), and FcRHT1 (Unigene0092206), as well as several structural genes involved in flavonoid biosynthesis, including *FcCHS* (Unigene0030769; Unigene0037171; Unigene0052596), *Fc4CL* (Unigene0068634), *FcDFR* (Unigene0030482), *FcPAL* (Unigene0044422), and *FcF3H* (Unigene0103205) (Fig. 9C). In the turquoise module, two key genes (*Fc4CL*, Unigene0077297; *FcPOD*, Unigene0112622) and two TFs (FcEIL3, Unigene0093001; FcSPA3, Unigene0057838) were identified (Supplementary Fig. 7A). Notably, in these three modules, both genes and transcription factors identified in the black and turquoise modules were downregulated, whereas those in the brown module were consistently upregulated (Fig. 9D-G, Supplementary Fig. 7B).

## Discussion

**Fig. 10:**
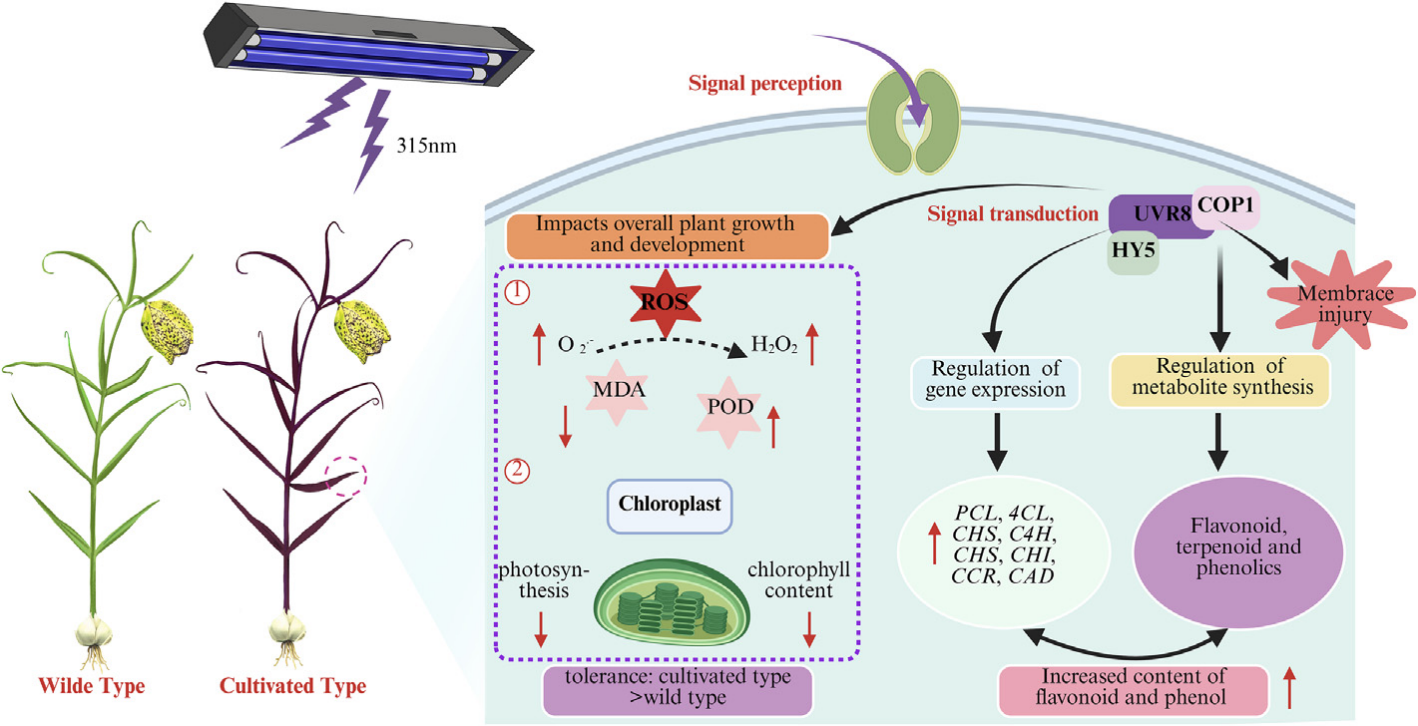
Proposed schematic model of molecular mechanisms underlying UV-B stress adaptation in *F. cirrhosa.* Comparative analysis of photosynthesis, leaf ultrastructure, redox state, and UV-absorbing compounds showed that the wild type *F. cirrhosa* is more sensitive and less tolerant to UV-B radiation than the cultivated type. The cultivated type exhibits enhanced UV-B tolerance, mediated by activation of the UVR8-COP1-HY5 signaling pathway and increased biosynthesis of phenolic compounds. These differences highlight the molecular and physiological basis of UV-B resistance. Such insights provide a foundation for breeding and conservation strategies to safeguard this valuable highland species.

UV-B radiation exerts wide-ranging effects on plants, from stimulating secondary metabolite production under mild exposure to causing severe damage to DNA, membranes, chloroplasts, and the photosynthetic system under high-intensity stress [14,[34], [35], [36], [37]]. Wild *F. cirrhosa*, which typically grow in shaded habitats dominated by *Sibiraea angustata* and *Rhododendron litangense*, experience low light intensity that restricts anthocyanin pathway activation. By contrast, cultivated plants exposed to full sunlight exhibit strong induction of anthocyanin biosynthesis, leading to purple pigmentation. Similar responses have been reported in *Fagopyrum tataricum* [38] and *Pisum sativum* [39], and UV-B is also known to promote anthocyanin biosynthesis [35,40]. Our previous revealed that key genes (*F3′H*, *ANS*, *DFR*) of the anthocyanin biosynthetic pathway were significantly upregulated only in cultivated types, with no activation detected in wild types [22]. Therefore, these results suggest that the leaf color transition from green (wild) to purple (cultivated) is primarily driven by environmentally induced anthocyanin biosynthesis rather than by fixed genetic differences in expressed genes. Although the cultivated type shows notable ecological adaptability, the molecular mechanisms underlying its quality development and UV-B stress tolerance remain unclear. In this study, the different inhibitory effects of UV-B on both types were analyzed by determining MDA content, ROS content, antioxidant enzyme activities, photosynthetic capacity, and degree of ultrastructural damage. To further reveal the molecular basis of the response of wild type and cultivated plants to UV-B stress, comparative multiomics studies were performed. Many DEGs and DAMs were linked to distinct KEGG pathways under UV-B stress (Fig. 10).

### Cultivated type exhibits stronger photosynthesis than the wild type under UV-B radiation

Photosynthesis is the most fundamental and complex physiological process in plants, and high photosynthetic efficiency promotes plant growth and metabolism [7]. In this study, UV-B radiation significantly inhibited the photosynthetic capacity of both wild and cultivated type plants; however, *Pn*, *Tr*, and *gsw* were more sensitive in the wild type than in the cultivated type (Fig. 1E). Decreased photosynthesis may result from both stomatal and non-stomatal limitations, with *Ci* used to distinguish between them [19]. Photosynthesis is considered stomatal-limited when *Pn*, *gsw*, and *Ci* decrease together, but non-stomatal-limited when *Pn* decreases while *Ci* remains stable or increases. In this study, *Ci* showed no significant changes under either treatment, suggesting that non-stomatal limitations—such as reduced chloroplast activity, Rubisco activity, and RuBP regeneration—were more pronounced. This finding is consistent with observations in *Schisandra chinensis* [41].

Chlorophyll is the main substance that absorbs light energy, which directly affects its use of light energy in plant photosynthesis [42]. In this study, the *SPAD* index of the wild plants was significantly reduced by UV-B radiation. In contrast, there was no significant difference between the cultivated types before and after treatment, possibly because the cultivated types developed resistance mechanisms to reduce chlorophyll damage under UV-B radiation (Fig. 1E). Chlorophyll fluorescence parameters can effectively reflect changes in actual photochemical efficiency and the degree of impairment of leaf photosystem II reaction centers due to UV-B stress [43,44]. Plants subject to photoinhibition slow electron movement by converting excess absorbed light into heat. *NPQ* is the process by which the photosystem II reaction center dissipates excess light energy as heat, and *qP* is the process of photochemical electron transfer [17]. In the present study, wild and cultivated type plants exposed to UV-B radiation exhibited a significant increase in *NPQ* and a significant decrease in *qP*, indicating that UV-B stress caused a decrease in the allocation of the light energy absorbed by photosystem II to the direction of photochemical reactions and an increase in the allocation of light energy to heat dissipation. This increased heat dissipation protects *F. cirrhosa* by preventing excess energy buildup in photosystem II. Subsequently, we found that *Fo* was significantly elevated in both types, and the *Fo* elevation was the result of damage to the membrane structure of the thylakoid and irreversible destruction of the photosystem II reaction center due to excess light energy [45], which again confirm the destruction of photosystem II in *F. cirrhosa* by UV-B stress. However, compared to the wild type, the cultivated type had a higher growth rate of *NPQ* and a lower rate of reduction of *qP* and growth rate of *Fo* under UV-B radiation, indicating that the inhibition of light and action of UV-B in the cultivated type was lower than that in the wild type. *ETR* was significantly increased in the cultivated type, which may be due to the rapid repair of damaged photosystem II during UV-B resistance in the cultivated type, allowing the photosystem to maintain its photosynthetic capacity (Fig. 1E). Meanwhile, our transcriptome analysis revealed that 23 DEGs were significantly upregulated in the cultivated type under UV-B stress, further supporting its ability to maintain superior photosynthetic capacity (Supplementary Fig. 3A).

### Under UV-B, the cellular ultrastructure of cultivated type was less damaged than that of the wild type

Susceptibility to UV-B radiation is evident in the degree of damage inflicted upon the leaf microstructure. SEM revealed that under UV-B stress, the number of attachments on the adaxial epidermis of leaves markedly increased and exhibited obvious fractures, suggesting that these structures may result from the accumulation of waxes and lignin (Fig. 2A). Such deposits have been shown to protect plant tissues by limiting non-stomatal water loss and gas exchange, shielding UV-B radiation, and influencing heat transfer [46,47]. Enhanced cuticular wax deposition under UV-B radiation has also been reported in several plant species, including *Cucumis sativus* [48] and *Zea mays* L [49]. Consistently, our transcriptome analysis showed that 12 DEGs involved in the cutin, suberine and wax biosynthesis pathway were upregulated in cultivated types after UV-B stress, in line with the SEM observations (Supplementary Fig. 3B). Therefore, this structural adaptation, together with enhanced heat dissipation and antioxidant defenses, may contribute to maintaining photosystem stability in the cultivated type. Stomata on the abaxial surface of the leaf epidermis were also significantly damaged and atrophied, and most of them appeared to be closed or opened to a very small extent. Under UV-B radiation, the cultivated type showed a smaller reduction in stomatal size and a significant increase in stomatal density compared to the wild type (Fig. 2B). This may be because the wild type is more susceptible to water loss under UV-B irradiation and needs to reduce leaf water loss by closing the stomata to reduce the transpiration rate, which is consistent with the stomatal exchange parameters. Furthermore, the morphology, size, organelle distribution, and cell wall structure of the leaf pulp cells of the wild and cultivated types changed significantly after UV-B treatment compared with CK, which might be the main cause of leaf greening and necrosis (Fig. 3B). Among these is the chloroplast, a key organelle in plant photosynthesis, the destructive effects of which can lead to disturbances in the structure and function of the thylakoid membrane and damage to the photosynthetic system, reducing photosynthetic efficiency and energy supply [7,50]. In the present study, the chloroplast morphology of the wild type and cultivated plants under UV-B stress changed significantly from shuttle-shaped to rounded, and the osmiophilic granules were heavily aggregated. However, compared with the cultivated type, the stroma-thylakoid of the wild type showed significant breakage, indicating that UV-B inhibited electron transfer in the photosystem II reaction center of both types; however, the cultivated type showed less inhibition than the wild type, which further validated the results of the chlorophyll fluorescence parameters (Fig. 3C).

### Cultivated type showed stronger antioxidant ability under UV-B radiation than did the wild type

Photosystem I and II reaction centers in chloroplasts are the main sites of ROS production. UV-B may trigger ROS formation mainly by inhibiting photosystems I and II and electron transport chain activity, as observed in studies on *Glycine* max L. cv. Punjab 1 and *Vicia faba* [51,52]. In the present study, significant increases in H_2_O_2_ and O_2_^–^ content were found in the wild type and cultivated plants under UV-B stress (Fig. 3D). However, wild type plants grew significantly faster than cultivated plants under UV-B exposure. This accelerated growth suggests ROS production may contribute to UV-B photodamage, which was more severe in wild type plants. To balance the overproduction of ROS, plants mitigate UV-B-induced oxidative damage through its own ROS scavenging system, in which POD is an important enzymatic antioxidant for plants to cope with UV-B radiation stress and is involved in a variety of physiological roles in plant tissues, including lignin biosynthesis, wound healing and pathogen defence [6,53,54]. In the present study, we found that POD activity increased only in the cultivated type, whereas there was no significant difference in the wild type (Fig. 3F). Additionally, UV-B radiation resists oxidative damage by regulating secondary metabolites in plants [36]. Among them, UV-absorbing substances, including flavonoids and phenolics, primarily accumulate in epidermal and leaf pulp cells, where they limit UV-B penetration into deeper leaf tissues and scavenge excess ROS, thereby providing protection against oxidative stress [55]. In this study, UV-B radiation significantly increased total flavonoid and phenol accumulation in both wild and cultivated plants, but the cultivated type exhibited a markedly higher growth rate than the wild type (Fig. 3E). These results suggest that the cultivated type could better activate the antioxidant system to resist oxidative damage. This was also reflected in the significant changes in MDA content (Fig. 3F). Although MDA is generally regarded as the end product of lipid peroxidation and a stress indicator, its decrease under UV-B treatment may suggest that the cultivated type enhanced its defense capacity by accumulating “sunscreen” flavonoids and rapidly inducing the activity of antioxidant enzymes such as POD, thereby effectively mitigating lipid peroxidation.

In summary, the comparative analysis of photosynthesis, ultrastructure, oxidation–reduction state, and UV-absorbing substances of both types of *F. cirrhosa* revealed that the wild type had a higher sensitivity than the cultivated type and lower tolerance to UV-B radiation than the cultivated type.

### UVR8 and transcription factors coordinately regulate the expression of flavonoid and lignin biosynthesis pathway genes in *F. Cirrhosa*

Recent studies of *Arabidopsis thaliana* have revealed that UV-B tolerance and adaptation in plants may be related to the expression of UVR 8-activated and its downstream TFs [29,56]. Under UV-B radiation, the inactive UVR8 homodimer in the cytoplasm can be converted into an active monomer and rapidly forms a COP1-UVR 8 complex with COP1 to improve the stability and activity of HY5, which in turn promotes UV-B-induced photomorphogenesis and regulates the expression of genes of various signalling pathways, especially the phenylpropanoid biosynthetic pathways, to regulate UV-B stress tolerance [57,58]. In the present study, we found that five DEGs encoding UVR8, three encoding COP1, and three encoding HY5 were significantly upregulated under UV-B stress in the cultivated type, whereas only one DEG encoding HY5 was significantly upregulated in the wild type. This indicates that UVR8-induced changes in transcript levels in *F. cirrhosa* leaves under UV-B irradiation are involved in UV-B tolerance and related gene expression, in conjunction with COP1 and HY5. However, the cultivated type exhibited a more positive regulatory mode (Fig. 5B-D). Second, RUP is a key negative regulator of the UVR8 pathway that promotes the recombination of UVR8 monomers into inactive homodimers, and its overexpression reduces UV-B-induced photomorphogenesis and impairs UV-B tolerance in plants [59,60]. In this study, there was no significant difference in RUP before and after treatment of the wild and cultivated types, suggesting that the ability of RUP to bind to UVR8 is weaker than that of COP1, confirming once again that *F. cirrhosa* resists UV-B stress with an active defense mechanism. UVR8 synergizes with the MYB and WRKY TFs families to regulate genes in the phenylpropanoid biosynthesis pathway, which is essential for metabolite modulation under UV-B radiation [61,62]. In the present study we identified 17 DEGs encoding the FcMYB family and 20 DEGs encoding the FcWRKY family as being involved in regulating the response of the cultivated type to UV-B stress, whereas only four DEGs encoding the FcMYB family and ten DEGs encoding the FcWRKY family were present in the wild type. These findings suggest that UV-B regulates genes in the UVR8 pathway and triggers downstream signaling by activating phenylpropanoid biosynthesis, thereby enhancing UV-B tolerance and promoting development in *F. cirrhosa* (Fig. 5E and F).

### Flavonoid and lignin biosynthesis pathways are associated with resistance to UV-B radiation in *F. Cirrhosa*

The lignin and flavonoid pathways are the two primary branches of the phenylpropanoid biosynthetic pathway, both of which provide precursors for their downstream metabolites through the transcriptional regulation of the *PAL*, *C4H* and *4CL* genes [63]. PAL is a rate-limiting enzyme that channels shikimate pathway metabolites into phenylpropanoid biosynthesis by converting phenylalanine to cinnamic acid. C4H, a cytochrome P450 monooxygenase, catalyzes the second step, hydroxylating cinnamic acid to p-coumaric acid. 4CL is the most important branch point involved in central phenylpropane biosynthesis in plants and catalyzes the conversion of p-coumaric acid to p-coumaroyl-CoA, a direct precursor for flavonoid or lignin biosynthesis [64,65]. In the cultivated type, these upstream genes were strongly induced under UV-B stress, thereby promoting the synthesis of downstream metabolites and supporting plant growth and development. This pattern is consistent with findings reported in *Withania somnifera* [66] and *Glycyrrhiza uralensis* [67]. However, the regulatory mechanisms underlying the different biosynthetic branches of the phenylpropanoid pathway, as well as the crosstalk between them, remain poorly understood. Therefore, we focused on elucidating the expression patterns of genes and metabolites in the lignin and flavonoid pathways of *F. cirrhosa* under UV-B stress (Fig. 8).

Flavonoids mainly accumulate in the epidermis and leaf pulp cells of plants and not only act as effective UV-B shielding filters but also possess strong ROS scavenging ability, which can effectively protect the plant photosystem from damage [13,68,69]. In plants, UV-B exposure induces the expression of genes involved in flavonoid biosynthesis, including *CHS*, *CHI*, *F3H*, *DFR* and *ANR* [70]. In this study, under UV-B radiation, upstream flavonoid biosynthesis genes (*FcCHS*, *FcCHI*, *FcF3H*) were significantly up-regulated in the cultivated type, while downstream genes (*FcDFR*, *FcANS*) were significantly up-regulated in the wild type. This suggests that different genes are differentially expressed in response to UV-B stimulation, owing to the type specificity of *F. cirrhosa*. Unexpressed genes downstream of the cultivated type may require a longer period of UV-B stress to reach maximal expression. UV-B radiation selectively stimulates the production of metabolites via the flavonoid biosynthetic pathway [36]. Xanthohumol, chrysin, galangin, pinocembrine, and naringenin in the leaves of the wild type and cultivated plants in the present study were the most sensitive to UV-B radiation, and all of them showed significant dynamic changes, except that the naringenin content was significantly reduced, whereas the rest of the contents were significantly increased. Consequently, from the current results, xanthohumol, chrysin, galangin and pinocembrine are to some extent UV-B protectors in wild type and cultivated type. Dihydroquercetin, a flavonol compound, is synthesized from naringenin and its hydroxylation can positively affect its antioxidant capacity [71]. However, we found that the dihydroquercetin content significantly decreased in the purple phenotype of *F. cirrhosa*, which may be attributed to the possible inhibition of the synthesis of the precursor compound naringenin, resulting in reduced production of dihydroquercetin. Luteolin is a potent ROS scavenger [72]. The significant increase in its content in the wild type also suggests that it may act as a specific UV-B protectant in the wild type. Subsequently, the significant increase in total phenolic and flavonoid contents of wild type and cultivated plants after UV-B stress provided a higher level of resistance, consistent with the results of studies on *Medicago sativa* L [73] and *Sinopodophyllum hexandrum* [74]. The increase in flavonoid content was attributed to UV-B-induced activation of flavonoid biosynthesis genes in *F. cirrhosa* leaves, which enhanced antioxidant activity and improved UV-B tolerance.

Phenylpropanoid-derived lignin polymers enhance plant structural stability and resilience against environmental stresses, such as UV-B radiation, and also contribute to water and nutrient transport [63]. Lignin biosynthesis involves two key steps: the production of monomeric lignin and its subsequent polymerization through free-radical coupling [75]. Specific enzymes such as *CCR*, *CAD*, *HCT*, *C3′H*, *COMT*, *CCoAOMT*, and *CSE* participate in monomer biosynthesis [76]. *CCR* and *CAD* convert p-coumaroyl-CoA into the simplest lignin monomer, p-coumaryl alcohol, whereas the biosynthesis of coniferyl alcohol additionally requires *C3′H*, *HCT*, *CSE*, *COMT*, *CCoAOMT*, and *4CL*. *F5H* and *COMT* are crucial for sinapyl alcohol formation [63]. *POD* functions both as an enzymatic antioxidant and as a key catalyst in the oxidative polymerization of monolignols into macromolecular lignin [77]. In our study, the cultivated type showed preferential induction of lignin-related genes, particularly those encoding *FcCCR*, *FcCAD*, *FcHCT*, *FcC3′H*, *FcCSE*, and *FcPOD*. This suggests that UV-B stress may stimulate the biosynthesis of p-coumaryl alcohol and coniferyl alcohol monomers in the cultivated type, followed by oxidative polymerization into H- and G-lignin. Such structural reinforcement likely reduces epidermal UV-B transmittance and provides additional protection. However, no lignin-related metabolites were detected, which may be due to limitations in the reference metabolite library.

### Roles of MYB and WRKY family TFs in regulating lignin and flavonoid biosynthesis

In plants, lignin and flavonoid biosynthesis is not only governed by structural genes but also tightly regulated by TFs. These TFs modulate the expression of one or more structural genes in the pathway by binding to *cis*-acting elements within their promoters, thereby controlling lignin and flavonoid production. Among them, MYB and WRKY are the most widely studied TFs families in plants. Numerous MYB factors have been confirmed to participate in the regulation of secondary metabolism, particularly in the biosynthesis of flavonoids and lignin [[78], [79], [80]]. For instance, in *Camellia sinensis*, MYBs regulate the expression of *F5′3′H*, *DFR*, and *LAR*, thereby altering flavonoid accumulation [81]. Similarly, GhMYB4 directly regulates the expression of *C4H* in the lignin and flavonoid pathways of *Gossypium hirsutum* under UV-B radiation, ultimately affecting anthocyanin biosynthesis [82]. In this study, FcMYB306 (Unigene0027420) and FcMYB90 (Unigene0059951) were identified as key positive regulators of *Fc4CL* (Unigene0068634), *FcDFR* (Unigene0030482), and *FcF3H* (Unigene0103205), which appear to promote flavonoid biosynthesis in *F. cirrhosa* under UV-B stress (Fig. 9E&F). In addition to MYBs, WRKY TFs also play essential roles in regulating lignin metabolism [83]. For example, Wang et al. demonstrated that overexpression of OsWRKY89 activates multiple UV-B responsive pathways, including lignin biosynthesis [84], while BrWRKY70 overexpression in *Brassica rapa* significantly upregulates lignin-related genes such as *AtCCoAOMT1*, *AtCOMT*, and *AtPOD* [85]. Consistently, in the present study, the expression profile of *FcCCoAOMT* (Unigene0102269) was closely aligned with members of the WRKY family, suggesting that FcWRKY may promote lignin biosynthesis through positive regulation of *FcCCoAOMT* (Fig. 9D&G). Beyond MYB and WRKY, NAC, ERF, and other TFs families also contribute to the regulation of lignin and flavonoid biosynthesis in *F. cirrhosa*. However, due to the large genome size of *F. cirrhosa*, the predicted regulatory functions of these candidate genes remain to be experimentally validated.

### Practical implications

Our findings provide insights into the molecular and metabolic mechanisms underlying UV-B tolerance in *F. cirrhosa*, which could be leveraged to improve UV-B resilience in crops. Beyond molecular breeding, cultivators can adopt practical strategies to mitigate UV-B stress, such as using UV-blocking greenhouse films or shading nets to reduce harmful radiation and maintain optimal light conditions for photosynthesis. Additionally, the key metabolites identified in this study could potentially be applied as foliar sprays to enhance UV-B tolerance, offering a complementary approach to protect crops under high UV-B exposure; these foliar application strategies will be further validated in future experiments.

## Conclusion

Our findings indicate that wild type *F. cirrhosa* is more sensitive and less tolerant to UV-B radiation than is the cultivated type. The cultivated plants demonstrated greater photosynthetic capacity, higher antioxidant enzyme activity, elevated levels of UV-absorbing substances (flavonoids and phenolics), and reduced ROS content (H_2_O_2_ and O_2_^–^). With this study we have developed a molecular model of UV-B resistance in *F. cirrhosa*. Upon exposure to UV-B radiation, the photoreceptor, UVR8, rapidly binds to COP1 to form a UVR8-COP1 complex. This complex stabilizes HY5 and interacts with FcMYB and FcWRKY transcription factor families to regulate target genes involved in lignin and flavonoid biosynthesis. In the cultivated type, galangin was identified as a specific UV-B protectant, whereas luteolin served as a protectant in the wild type. These results indicated that the cultivated type had a stronger resistance to UV-B radiation. We also elucidated the mechanism of UV-B radiation resistance and ecological adaptability of the cultivated type, which is important for the promotion and demonstration of artificial domestication cultivation and the introduction of planting in low-altitude areas, as well as a reference for the selection and breeding of new varieties of *F. cirrhosa* and standardized cultivation of this important medicinal plant.

Compliance with Ethics Requirements.

We the undersigned declare that this manuscript entitled “Physiological and multi-omics insights into ultraviolet B-induced stress adaptation in *Fritillaria cirrhosa* native to the Qinghai-Tibet Plateau“ isoriginal, has not been published before and is not currently being considered forpublication elsewhere. We confirm·that the manuscript has been read and approved by allnamed authorsand that there are no other persons who satisfied the criteria for authorship·but are notlisted. We further confim·that the order of authors listed in the manuscript has beenapproved by allofus. This paper does not involve animal experiments or clinical trials, so there are no ethical issues.

Data Availability Statement.

We have uploaded the RNA sequencing data to NCBI and the link is https://www.ncbi.nlm.nih.gov/sra/PRJNA1146953. The data sets supporting the results of this article are included within the article and supplementary table.

## CRediT authorship contribution statement

**Zemin Yang:** Investigation, Methodology, Data curation, Writing – original draft. **Dan Gao:** Validation, Supervision, Funding acquisition, Writing – review & editing. **Ye Wang:** Software, Writing – review & editing. **Haitao Liu:** Writing – review & editing. **Yuhan Wu:** Investigation, Software. **Haobo Zhang:** Investigation, Software. **Haiqing Wang:** Investigation, Resources. **Xusheng Gao:** Software. **Jialu Wang:** Investigation. **Yonggang Wang:** Software. **Huigan Xie:** Resources. **Shaobing Fu:** Resources. **Xiwen Li:** Conceptualization, Supervision, Funding acquisition, Writing – review & editing.

## Declaration of competing interest

All authors declare that the research was conducted in the absence of any commercial or financial relationships that could be construed as a potential conflict of interest.
