# Supplementary material for: Physiological and multi-omics insights into ultraviolet B-induced stress adaptation in Fritillaria cirrhosa native to the Qinghai-Tibet Plateau
Source: J Adv Res. 2025 Oct 26;85:155–74. doi: 10.1016/j.jare.2025.10.049 (PMC13316609; doi:10.1016/j.jare.2025.10.049)
Supplement: Supplementary Data 1 [file mmc1.docx]

Manuscript for Journal of Advanced Research

**Physiological and multi-omics insights into ultraviolet B-induced stress adaptation in *Fritillaria cirrhosa* native to the Qinghai-Tibet Plateau**

Zemin Yang ^a,b^, Dan Gao ^a*^, Ye Wang ^c^, Haitao Liu ^d^, Yuhan Wu ^a,e^, Haobo Zhang ^a,e^, Haiqing Wang ^j^, Xusheng Gao ^e^, Jialu Wang ^a^, Yonggang Wang ^f^, Huigan Xie ^j^, Shaobing Fu ^j^, Xiwen Li ^a,d*^

^a^ State Key Laboratory for Quality Ensurance and Sustainable Use of Dao-di Herbs, Institute of Chinese Materia Medica, China Academy of Chinese Medical Sciences, Beijing 100700, China;

^b^ State Key Laboratory of Phytochemistry and Natural Medicines, Kunming Institute of Botany, Chinese Academy of Sciences, Kunming, 650201, China;

^c^ Institute of Traditional Chinese Medicine Health Industry, China Academy of Chinese Medical Sciences, Nanchang 330000, China;

^d^ Institute of Medicinal Plant Development, Chinese Academy of Medical Sciences & Peking Union Medical College, Beijing, 100193, China;

^e^ College of Chinese Medicinal Materials, Jilin Agricultural University, Changchun 130118, China;

^f^ School of Life Science and Engineering, Lanzhou University of Technology, Lanzhou 730050, China

^j^ Nin Jiom Medicine Manufactory (Hong Kong) Limited, Hong Kong 999077, China;

* Corresponding author:

Prof. Xiwen Li and Dr. Dan Gao, Institute of Chinese Materia Medica, China Academy of Chinese Medical Sciences, Beijing 100700, China

Telephone/fax: +86–84084107; E–mail: xwli@icmm.ac.cn; [dgao@icmm.ac.cn.](mailto:dgao@icmm.ac.cn.)**Detailed Catalog**

**1.Supplementary Method 1**

**2.Supplementary Method 2**

**3.Supplementary Method 3**

**4.Supplementary Method 4**

**5.Supplementary Method 5**

**6.Supplementary Figure 1-7**

**1. Supplementary Method 1**

**Experimental soil parameters**

Organic carbon (30.39 g/kg), total nitrogen (2.88 g/kg), effective phosphorus (83.11 mg/kg), total phosphorus (1.38 g/kg), total potassium (21.66 g/kg), alkaline nitrogen decomposition (171.75 mg/kg), quick-acting potassium (234.00 mg/kg), and moisture content (28.78%).

**2. Supplementary Method 2**

**Measurement of photosynthetic parameters**

The photosynthetic parameters of both types *F. cirrhosa* under the CK and UV-B treatments were measured using an LI-6800 portable photosynthesis system (LI-COR Biosciences, Lincoln, NE, USA). The third pair of apical mature leaves was selected to ensure complete filling of the 2 cm² leaf chamber, avoiding vein entrapment during clamping. Measurements were conducted between 8:30 and 11:30 AM under controlled conditions: photosynthetic photon flux density (PPFD) was set to 1,200 μmol·m⁻²·s⁻¹, chamber CO₂ concentration was maintained at 400 μmol·mol⁻¹ via compressed gas, relative humidity was 55%, and airflow rate was 500 μmol·s⁻¹. In each treatment group, five plants were randomly selected, and three biological replicates were used for each plant. The measured parameters included the net photosynthetic rate (*Pn*), intercellular CO_2_ concentration (*Ci*), transpiration rate (*Tr*), stomatal conductance (*gsw*), and other gas exchange parameters. Additionally, the quantum yield of photosystem II (*ΦPSII*), non-photochemical quenching coefficient (*NPQ*), photochemical fluorescence quenching coefficient (*qP*), electron transfer rate (*ETR*), and other light parameters of chlorophyll fluorescence were measured. To acclimatize the leaves to darkness, they were covered with tin foil for 20 min. The original fluorescence yield (*Fo*), maximal fluorescence yield (*Fm*), and maximum photochemical efficiency of PSII (*Fv/Fm*) were determined.

**3. Supplementary Method 3**

**Scanning electron microscopy and transmission electron microscope methods**

Leaf tissues treated with CK and UV-B were meticulously fixed with 2.5% glutaraldehyde at 4°C for 12 hours to preserve their structural integrity. Subsequently, the samples were fixed in a mixed solution of 1% OsO_4_ and 2% K_3_[Fe(CN)_6_] for 1.5 hours and washed repeatedly with deionized water. To ensure optimal sample dehydration, the samples were subjected to a series of gradient dehydration steps using acetone solutions of varying concentrations. Following dehydration, the leaf and bulb tissues were sectioned into ultrathin slices with a thickness of 70–90 nm using a state-of-the-art ultrathin microtome (Wetzlar, Germany). Thin sections were collected on copper mesh and stained with uranyl acetate and lead citrate. The morphology and ultrastructure of the samples were characterized using an HT7800 biological transmission electron microscope (TEM) (Tokyo, Japan), which provided high-resolution images that enabled detailed observations [1]. The elemental compositions of the samples were analyzed using an advanced JED-2300T Energy Scattering X-ray fluorescence spectrometer (Tokyo, Japan), which allowed the quantification and identification of the elements present in the samples.

It is important to note the differences between scanning electron microscopy (SEM) and TEM. For SEM, the samples were fixed with 2.5% glutaraldehyde, rinsed with phosphate buffer, and fixed in a 1% OsO_4_ solution. Following a series of ethanol dehydration steps, the samples were dried, fixed onto sample stages, and coated with a thin Pt layer. SEM was performed using a Regulus 8100 Bio-SEM (Tokyo, Japan). Scanning electron microscopy results were analyzed for stomatal characteristics using ImageJ software [2].

**4. Supplementary Method 4**

**Transcriptomic methods**

After harvesting the fresh leaves of *F. cirrhosa*, we washed them with ultrapure water and placed them in RNase-free cryopreservation tubes. The tubes were sealed, submerged in liquid nitrogen for 30 minutes, and then stored in a -80°C refrigerator. Total RNA was extracted following the manufacturer’s instructions of a Trizol kit (Invitrogen, Carlsbad, CA, USA). RNA quality was assessed using an Agilent 2100 Bioanalyzer (Agilent Technologies, Palo Alto, CA, USA) and integrity was checked by RNase-free agarose gel electrophoresis. RNA purity was assessed using a Nanodrop microspectrophotometer (Thermo Fisher Scientific, MA, Waltham, USA), and only samples meeting quality standards were selected for RNA-seq analysis. Each sample used 1 µg of RNA as input material. Sequencing libraries were constructed using the Hieff NGS® Ultima Dual-mode mRNA Library Preparation Kit (Shanghai Yeisen Biotechnology Co., Ltd.) according to the manufacturer's protocols, with three biological replicates per treatment, each replicate consisting of a mixture of three to six leaves from a plant. Upon completion of library quality assessment utilizing the High Sensitivity DNA assay Kit (Agilent Technologies, Palo Alto, CA, USA), high-throughput sequencing was carried out on the Illumina novaseq 6000 sequencing platform by Guangzhou Kidio Biotechnology Co Ltd (Guangzhou, China). Subsequently, low-quality reads were filtered out using fastp (v0.18.0) to obtain high-quality clean reads [3], which were then de novo assembled employing Trinity (v2.11.0) [4]. After assembly, we employed the BLASTx program with an E-value of 1e-5 to annotate several databases, including the NCBI Non-Redundant Protein (Nr), Swiss-Prot, protein family (Pfam), Kyoto Encyclopedia of Genes and Genomes Pathways (KEGG), Gene Ontology (GO), and COG/KOG databases. Transcripts Per Kilobase of exon model per Million mapped reads (TPM) values were determined utilizing the RSEM software to quantify the abundance and variations in gene expression [5]. Subsequently, the edgeR software was utilized to identify significantly different genes between two phenotypic CK and UV-B treatments based on the conditions of FDR < 0.05 and |log_2_Fold Change| > 1 [6].

Gene Set Enrichment Analysis (GSEA) [7] combined with the Molecular Signatures Database (MSigDB) [8] was performed to identify differentially enriched gene sets associated with specific pathway annotations between two experimental groups. Briefly, the gene expression matrix was processed, and genes were ranked using the Signal2Noise metric. Enrichment scores (ES) and corresponding p-values were calculated with default software parameters, followed by false discovery rate (FDR) correction to account for multiple hypothesis testing.

**5. Supplementary Method 5**

**Metabolomic methods**

A 100 mg sample was taken from each of the two phenotypes of CK- and UV-B-treated leaf tissues. The samples were ground with liquid nitrogen and mixed with 1 mL of a pre-cooled aqueous methanol-acetonitrile solution (2:2:1, v/v). After sonication at low temperature for 60 minutes, the mixture was centrifuged at 14,000g for 20 minutes at 4°C. The resulting supernatant, equivalent to 50 mg of the sample, was vacuum-dried. For mass spectrometry analysis, 100 μL of an acetonitrile-water solution (1:1, v/v) was added, followed by centrifugation at 14,000g and 4°C for 15 minutes, and the supernatant was collected for analysis. Metabolomics analyses were conducted by Gene Denovo (Guangzhou, China) using high-performance liquid chromatography-tandem mass spectrometry (LC-MS/MS). Each sample set was analyzed in triplicate. Aliquots from each experimental group were pooled to create a quality control (QC) sample. Instrumental parameters can be found in **Table 1**. Multivariate statistical analysis using VIP values from OPLS-DA, along with univariate analysis of T-test *P*-values, was employed to identify significantly different metabolites between comparison groups. The screening criteria were VIP ≥ 1 and T-test *P* < 0.05 in the OPLS-DA model.

| Table 1 Instrumental parameters | | | |
| --- | --- | --- | --- |
| **Chromatographic conditions** |  | | |
| Column | Xselect HSS T3, 2.5 μm, 2.1×150 mm | | |
| Mobile phase | A: 0.1% formic acid-water | B: 0.1% formic acid acetonitrile | |
| Column temperature | 50℃ | | |
| flowrate | 0.4 mL/min | | |
| Gradientelution | | | |
| 1 | 0 min | 98%A | 2%B |
| 2 | 2 min | 98%A | 2%B |
| 3 | 15 min | 0%A | 100%B |
| 4 | 17 min | 0%A | 100%B |
| 5 | 17.1 min | 98%A | 2%B |
| 6 | 20 min | 98%A | 2%B |
| **Mass Spectrometry Conditions** |  |  |  |
| Positive ionisation mode |  | Negative ionization mode |  |
| Curtain Gas | 35psi | Curtain Gas | 35psi |
| Collision Gas | Medium | Collision Gas | Medium |
| IonSpray Voltage | 5500V | IonSpray Voltage | －4500V |
| Temperature | 550℃ | Temperature | 550℃ |
| Ion Source Gas | 1:60 | Ion Source Gas | 1:60 |
| Ion Source Gas | 2:60 | Ion Source Gas | 2:60 |

1. **Supplementary figure legends**

**Supplementary Figure 1:** Metabolite chromatograms in both types. CKW - Wild type control; UVW - Wild type treatment; CKC - Cultivated type control; UVC - Cultivated type treatment, similar to the following image.

**Supplementary Figure 2:** Transcriptome analysis reveals genotype-specific responses to UV-B in wild and cultivated *F. cirrhosa*. (A) PCA of transcriptome gene expression, showing differences between samples based on DEGs. Volcano plots (B) and clustering heat maps (C) highlighting transcriptional responses to UV-B in the two types, showing a stronger response in the cultivated type than in the wild type. (D) GO enrichment analyses of *F. cirrhosa* show the shared BP terms between the cultivated and wild types.

**Supplementary Figure 3:** Supplementary Figure 3 Heat maps of DEGs related to photosynthesis (A) and lignin, tannin, and wax biosynthesis (B) in wild and cultivated F. cirrhosa under UV-B stress.

**Supplementary Figure 4** Correlation analysis of flavonoids and antioxidant activity parameters (POD, MDA, O_2_^-^ and H_2_O_2_). * or * * indicates a significant difference at the 0.05 or 0.01 level, respectively.

**Supplementary Figure 5** The gene expression was determined by RT-qPCR. Data are expressed as Mean ± Se (n = 3). The different * *P* < 0.05 at the top of the bars indicate significant differences between the groups by one-way ANOVA

**Supplementary Figure 6** Co-expression network modules identified by WGCNA. (A) Determination of the soft-thresholding power (β) in WGCNA based on the adjacency matrix. (B) Hierarchical clustering dendrogram of genes in WGCNA. The upper part shows the gene dendrogram, while the lower part displays the assigned modules, with modules of the same color representing similar co-expression patterns.

**Supplementary Figure 7** (A) Associated networks of differentially expressed structural genes and TFs involved in lignin and flavonoid biosynthesis. (B) Expression patterns of genes and TFs in the black and brown modules, showing downregulation in the turquoise modules

**Supplementary Figure 1** Metabolite chromatograms in both types. CKW - Wild type control; UVW - Wild type treatment; CKC - Cultivated type control; UVC - Cultivated type treatment, similar to the following image.

**Supplementary Figure 2:** Transcriptome analysis reveals genotype-specific responses to UV-B in wild and cultivated *F. cirrhosa*. (A) PCA of transcriptome gene expression, showing differences between samples based on DEGs. Volcano plots (B) and clustering heat maps (C) highlighting transcriptional responses to UV-B in the two types, showing a stronger response in the cultivated type than in the wild type. (D) GO enrichment analyses of *F. cirrhosa* show the shared BP terms between the cultivated and wild types.

**Supplementary Figure 3** Heat maps of DEGs related to photosynthesis (A) and lignin, tannin, and wax biosynthesis (B) in wild and cultivated *F. cirrhosa* under UV-B stress.

**Supplementary Figure 4** Correlation analysis of flavonoids and antioxidant activity parameters (POD, MDA, O_2_^-^ and H_2_O_2_). * or * * indicates a significant difference at the 0.05 or 0.01 level, respectively.

**Supplementary Figure 5** The gene expression was determined by RT-qPCR. Data are expressed as Mean ± SE (n = 3). The different * *P* < 0.05 at the top of the bars indicate significant differences between the groups by one-way ANOVA

**Supplementary Figure 6** Co-expression network modules identified by WGCNA. (A) Determination of the soft-thresholding power (β) in WGCNA based on the adjacency matrix. (B) Hierarchical clustering dendrogram of genes in WGCNA. The upper part shows the gene dendrogram, while the lower part displays the assigned modules, with modules of the same color representing similar co-expression patterns.

**Supplementary Figure 7** (A) Associated networks of differentially expressed structural genes and TFs involved in lignin and flavonoid biosynthesis. (B) Expression patterns of genes and TFs in the black and brown modules, showing downregulation in the turquoise modules

**References:**

[1] Wang, J., Chen, J., Zhang, X., Feng, X., Li, X.,Physiological and transcriptional responses to heat stress in a typical phenotype of *Pinellia ternata*. Chin J Nat Med 2023; 21:243-252. doi: https://doi.org/10.1016/S1875-5364(23)60433-9

[2] Yang, Z., Wang, J., Wang, W., Zhang, H., Wu, Y., Gao, X., Gao, D., Li, X.,Physiological, cytological and multi-omics analysis revealed the molecular response of Fritillaria cirrhosa to Cd toxicity in Qinghai-Tibet Plateau. J Hazard Mater 2024; 472:134611. doi: https://doi.org/10.1016/j.jhazmat.2024.134611

[3] Chen, S., Zhou, Y., Chen, Y., Gu, J.,fastp: an ultra-fast all-in-one FASTQ preprocessor. Bioinformatics 2018; 34:i884-i890. doi: https://doi.org/10.1093/bioinformatics/bty560

[4] Grabherr, M.G., Haas, B.J., Yassour, M., Levin, J.Z., Thompson, D.A., Amit, I., Adiconis, X., Fan, L., Raychowdhury, R., Zeng, Q., Chen, Z., Mauceli, E., Hacohen, N., Gnirke, A., Rhind, N., di Palma, F., Birren, B.W., Nusbaum, C., Lindblad-Toh, K., Friedman, N., Regev, A.,Full-length transcriptome assembly from RNA-Seq data without a reference genome. Nat Biotechnol 2011; 29:644-652. doi: https://doi.org/10.1038/nbt.1883

[5] Li, B., Dewey, C.N.,RSEM: accurate transcript quantification from RNA-Seq data with or without a reference genome. Bmc Bioinformatics 2011; 12:323. doi: https://doi.org/10.1186/1471-2105-12-323

[6] Robinson, M.D., McCarthy, D.J., Smyth, G.K.,edgeR : a Bioconductor package for differential expression analysis of digital gene expression data. Bioinformatics 2010; 26:139-140. doi: https://doi.org/10.1093/bioinformatics/btp616

[7] Subramanian, A., Tamayo, P., Mootha, V.K., Mukherjee, S., Ebert, B.L., Gillette, M.A., Paulovich, A., Pomeroy, S.L., Golub, T.R., Lander, E.S., Mesirov, J.P.,Gene set enrichment analysis: a knowledge-based approach for interpreting genome-wide expression profiles. Proc Natl Acad Sci U S A 2005; 102:15545-15550. doi: https://doi.org/10.1073/pnas.0506580102

[8] Van der Auwera, G.A., Carneiro, M.O., Hartl, C., Poplin, R., Del Angel, G., Levy Moonshine, A., Jordan, T., Shakir, K., Roazen, D., Thibault, J., Banks, E., Garimella, K.V., Altshuler, D., Gabriel, S., DePristo, M.A.,From FastQ Data to High‐Confidence Variant Calls: The Genome Analysis Toolkit Best Practices Pipeline. Current Protocols in Bioinformatics 2013; 43:10. doi: https://doi.org/1002/0471250953.bi1110s43
